# Progress towards onchocerciasis elimination in the participating countries of the African Programme for Onchocerciasis Control: epidemiological evaluation results

**DOI:** 10.1186/s40249-016-0160-7

**Published:** 2016-06-27

**Authors:** Afework H. Tekle, Honorath G. M. Zouré, Mounkaila Noma, Michel Boussinesq, Luc E. Coffeng, Wilma A. Stolk, Jan H. F. Remme

**Affiliations:** African Programme for Onchocerciasis Control, Ouagadougou, Burkina Faso; Institut de Recherche pour le Développement (IRD), Montpellier, France; Department of Public Health, Erasmus MC, University Medical Center Rotterdam, Rotterdam, The Netherlands; 120 rue des Campanules, Ornex, France

**Keywords:** Onchocerciasis, Ivermectin, APOC, Elimination, Community-directed treatment, ONCHOSIM

## Abstract

**Background:**

The African Programme for Onchocerciasis Control (APOC) was created in 1995 to establish community-directed treatment with ivermectin (CDTi) in order to control onchocerciasis as a public health problem in 20 African countries that had 80 % of the global disease burden. When research showed that CDTi may ultimately eliminate onchocerciasis infection, APOC was given in 2008 the additional objective to determine when and where treatment can be safely stopped. We report the results of epidemiological evaluations undertaken from 2008 to 2014 to assess progress towards elimination in CDTi areas with ≥6 years treatment.

**Methods:**

Skin snip surveys were undertaken in samples of first-line villages to determine the prevalence of *O. volvulus* microfilariae. There were two evaluation phases. The decline in prevalence was evaluated in phase 1A. Observed and model-predicted prevalences were compared after correcting for endemicity level and treatment coverage. Bayesian statistics and Monte Carlo simulation were used to classify the decline in prevalence as faster than predicted, on track or delayed. Where the prevalence approached elimination levels, phase 1B was launched to determine if treatment could be safely stopped. Village sampling was extended to the whole CDTi area. Survey data were analysed within a Bayesian framework to determine if stopping criteria (overall prevalence <1.4 % and maximum stratum prevalence <5 %) were met.

**Results:**

In phase 1A 127 665 people from 639 villages in 54 areas were examined. The prevalence had fallen dramatically. The decline in prevalence was faster than predicted in 23 areas, on track in another 23 and delayed in eight areas. In phase 1B 108 636 people in 392 villages were examined in 22 areas of which 13 met the epidemiological criteria for stopping treatment. Overall, 32 areas (25.4 million people) had reached or were close to elimination, 18 areas (17.4 million) were on track but required more years treatment, and in eight areas (10.4 million) progress was unsatisfactory.

**Conclusions:**

Onchocerciasis has been largely controlled as a public health problem. Great progress has been made towards elimination which already appears to have been achieved for millions of people. For most APOC countries, nationwide onchocerciasis elimination is within reach.

**Electronic supplementary material:**

The online version of this article (doi:10.1186/s40249-016-0160-7) contains supplementary material, which is available to authorized users.

## Multilingual abstracts

Please see Additional file 1 for translations of the abstract into the six official working languages of the United Nations.

## Background

Onchocerciasis has been an important public health problem in tropical Africa, Latin America, and the Yemen with over 40 million people infected before the launch of large-scale control and an at-risk population of over 160 million of which more than 99 % live in Africa [1–4]. Thirty-one countries in Africa - 20 which are participating countries of the African Programme for Onchocerciasis Control (APOC) [5] and 11 which were members of the previous Onchocerciasis Control Programme in West Africa (OCP) [6] - were known or suspected to be endemic for onchocerciasis [1]. Human onchocerciasis is caused by the filarial parasitic nematode *Onchocerca volvulus* which is transmitted through repeated bites by blackflies of the genus *Simulium*. It is the world’s second leading infectious cause of blindness and the disease is also known as river blindness because the blackfly that transmits the parasite lives and breeds near fast-flowing streams and rivers. In addition to ocular complications, onchocerciasis causes skin disease, including unsightly skin lesions and debilitating itching, excess mortality among highly infected people and it is also a major risk factor for epilepsy and nodding syndrome [7–10]. Due to its negative impact on health, social well-being and productivity, onchocerciasis perpetuates poverty and under-development [11].

The Onchocerciasis Control Programme in West Africa (OCP) started large scale control of onchocerciasis in 1974 through vector control, using aerial spraying of environmental safe larvicides [6]. This strategy was later complemented by ivermectin mass treatment of the at-risk population following the donation of ivermectin in 1987 [12]. At its closure by the end of 2002, OCP had successfully eliminated the disease as a public health and socio-economic problem in all 11 OCP countries except Sierra Leone where control activities were interrupted because of civil unrest [13]. Ivermectin proved to be very safe and highly effective against microfilariae (mf) which cause the severe manifestations of the disease [14, 15], and community-directed treatment with ivermectin (CDTi) was proven to be an effective and sustainable strategy for annual treatment of the eligible population in endemic communities [16, 17]. CDTi was adopted as the mainstay of onchocerciasis control in APOC-supported countries. APOC was initiated in 1995 with the ultimate goal of controlling onchocerciasis as a public health problem in those African countries not covered by the OCP and which had more than 80 % of the global burden of onchocercal disease [4, 5, 18].

The APOC partnership has helped onchocerciasis endemic countries to successfully extend CDTi coverage from 1.5 million in 1997 to over 112 million people in 2014, when ivermectin reached over 180 000 communities in Africa [19]. It has enabled treatment of approximately 76 % of the total population at risk including several countries affected by security issues. Annual CDTi has conferred enormous clinical benefits, averting the loss of 2 million Disability-adjusted Life Years every year at a cost of only US$27 per Disability-adjusted Life Year averted, making it very cost-effective [20].

Both OCP and APOC were set up with the objective to control the disease as a public health problem rather than to achieve elimination of the infection and transmission. At the time it was considered doubtful that elimination could be achieved with ivermectin alone and a conference on the eradicability of onchocerciasis concluded that elimination would be possible in most of the Americas where onchocerciasis foci are often small and circumscribed, and several (though not all) vector species are relatively inefficient, but that there remained considerable uncertainty as to whether ivermectin treatment could ever achieve sustained interruption of transmission in Africa where onchocerciasis is endemic over vast areas and where all vectors are highly efficient [21].

However, subsequent longitudinal studies were able to show the feasibility of elimination of onchocerciasis in some African setting using only ivermectin treatment. In early 2000s, a detailed review of the impact of ivermectin treatment on onchocerciasis infection in the OCP countries showed that prevalence of infection had fallen to very low levels after 10–12 years of treatment in onchocerciasis foci in the Western extension area where there had never been vector control [22]. In 2005 a longitudinal study was started in three initially hyperendemic onchocerciasis foci in Mali and Senegal where ivermectin had been given for 15–17 years at annual (two foci) and 6-monthly (one focus) intervals. The aim of the study was to undertake a detailed assessment of residual levels of infection and transmission and to test whether ivermectin treatment could be safely stopped. In all three foci the epidemiological and entomological indicators were below or close to provisional thresholds [23]. Treatment was then stopped and follow-up data over a period of 3 to 5 years showed no evidence of new infection or transmission [24]. This study provided the first evidence that annual or 6-monthly ivermectin treatment can eliminate onchocerciasis infection and transmission, and that treatment can be safely stopped. Subsequent evidence from epidemiological evaluations in APOC countries also showed that infection levels had fallen to insignificant levels in some areas. Parasitological surveys based on skin snips examination undertaken in 2008 in two onchocerciasis endemic foci with annual ivermectin treatment in Kaduna State, Nigeria showed that the prevalence of mf was zero in all 27 surveyed villages [25].

In response to these new findings, the Joint Action Forum of APOC approved in 2008 an additional objective for the programme “to determine when and where ivermectin treatment can be safely stopped and to provide guidance to countries on preparing to stop ivermectin treatment where feasible”.

An international group of experts was convened in early 2009 to review the state-of-the-art and identify critical issues for onchocerciasis elimination in Africa [26]. The experts defined onchocerciasis elimination operationally as “the reduction of onchocerciasis infection and transmission to the extent that interventions can be stopped, but post-intervention surveillance is still necessary”. They recommended that APOC proceed gradually, targeting elimination where considered feasible and undertaking a critical evaluation of the epidemiological and operational situation in countries before adopting the goal of elimination. Building on the report of the expert group, APOC developed a conceptual and operational framework of onchocerciasis elimination that was further refined by the Technical Consultative Committee of APOC [27]. The conceptual framework document includes a description of the main operational phases in elimination and the evaluation methods and indicators to be used in each phase.

Following the approval of the new objective on onchocerciasis elimination, APOC has conducted a series of epidemiological evaluation surveys from 2008 to date. This article presents the results of the epidemiological evaluation surveys and a comprehensive analysis of the survey data collected between 2008 and 2015 in 12 APOC countries, showing the decline in onchocerciasis infection levels and estimates of the number of people freed from the risk of onchocerciasis infection. As APOC closed on December 31, 2015, this paper will also serve to indicate the status of all CDTi projects evaluated in the last 6–7 years and provide a basis for future decision making by the endemic countries on where and when to stop ivermectin treatment.

## Methods

### Conceptual framework of onchocerciasis elimination

**Fig. 1:**
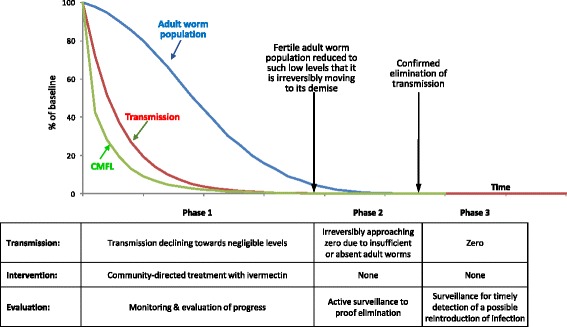
Conceptual framework for onchocerciasis elimination

Figure 1 illustrates APOC’s conceptual framework for onchocerciasis elimination [27]. After the first round of ivermectin treatment in an onchocerciasis focus, the intensity of infection in the community (expressed using the community microfilarial load, CMFL [28]) declines dramatically, and this translates into a significant drop in transmission. After each subsequent treatment round, the mean microfilarial load is further reduced and the annual level of transmission continues to decline. The adult worm population also shows a decline, although much more slowly, due to natural or treatment-induced death or sterilisation of old worms. This continues till the fertile adult worm population has been reduced to such low levels that it will move irreversibly to its extinction, even without further ivermectin treatment. The parasite density is said to have fallen below its “breakpoint” and ivermectin treatment can be stopped, signalling the end of the intervention phase 1. The concept of a breakpoint is operationally important: it means that infection and transmission do not have to be completely zero before treatment can be safely stopped. Breakpoints are predicted by models [29, 30] and confirmed empirically: in the proof-of-concept study in Mali and Senegal there were still several mf positive people in each of the three river basins but when treatment was stopped, there was no renewed transmission and infection [24]. The duration of phase 1 varies according to initial endemicity level and treatment coverage. The evaluation in this phase has two sequential objectives: (i) to evaluate the progress towards elimination by assessing the decline in infection levels in the human population towards provisional thresholds for elimination (evaluation phase 1A), and (ii) to confirm, using both parasitological and entomological indicators, that the provisional threshold has been reached and that treatment can be safely stopped (evaluation phase 1B at the end of phase 1).

In phase 2 the parasite numbers in the human and vector populations are so low that any residual transmission is expected to be insufficient for the parasite population to survive on the long term. Active surveillance including both entomological and epidemiological indicators is needed to make sure that there is no recrudescence of the parasite population and transmission. If no recrudescence is detected over a period of at least 3 years, the area is expected to have met or to be moving inevitably to meet the general definition of elimination of an infectious disease: ‘reduction to zero of the incidence of infection caused by a specific agent in a defined geographical area as a result of deliberate efforts; continued measures to prevent reestablishment of transmission are required’ [31]. The area then moves into phase 3 with routine surveillance to detect possible reintroduction of infection from other areas where the infection still occurs.

### Evaluation areas

In this article we report the results of epidemiological evaluations undertaken during phase 1. Since its inception, APOC has provided support to the establishment of sustainable annual CDTi in 106 CDTi projects that covered nearly all endemic areas in the APOC countries where onchocerciasis was a public health problem. Between 2008 and 2015, epidemiological evaluations were undertaken in 52 CDTi projects which had benefited from at least 6 years of annual ivermectin treatment. Most of these CDTi projects cover the onchocerciasis endemic area within the boundaries of an administrative unit of the health system that is responsible for the local implementation of CDTi, usually the health district, and where treatment was introduced at approximately the same time in all villages. Most of these projects were considered sufficiently homogeneous in terms of epidemiology and intervention history to include them as evaluation areas for the current analysis. However, in two countries, Chad and the Central African Republic, there is only one CDTi project for the whole country covering a wide range of endemic areas over a distance of 700 and 1400 km respectively and with many years difference in the start of treatment. The CDTi projects for these two countries were therefore divided into subprojects according to implementation unit of the health system, and these subprojects were used as evaluation areas in the current study. Furthermore, there were two situations (Edo and Ondo States in Nigeria and Keffa, Sheka and Bench-Maji zones in Ethiopia) where the evaluation was done in an endemic focus that ran across the border of neighbouring CDTi projects and these projects were combined into one evaluation area. In all other cases, the evaluation area corresponds to the CDTi project area, as defined by APOC.

Data for the target population and number of people treated in each evaluation area were obtained from the annual technical reports from each CDTi project to APOC. Treatment coverage was calculated as the percentage of the total population (≥0 years old) in the CDTi project area that was treated in a given year. For project areas where mass drug administration with ivermectin had been undertaken during years before the establishment of the APOC project an attempt was made to collect data on start year of treatment, number of years of treatment and treatment coverage from the Ministry of health and/or a non-governmental organisation that supported those early treatments. However, these data are less complete than those for the APOC period. A treatment coverage of less than 60 % was considered inadequate and years with such low coverage were not taken into account in the calculation of the number of years of ivermectin treatment of an evaluation area.

### Phase 1A evaluation

#### Objective

This is the evaluation activity for most of the phase 1 period. It involves parasitological surveys after at least 6 years of ivermectin treatment to assess the remaining levels of *O. volvulus* infection in a sample of communities from an endemic focus and to compare the survey results with the predicted prevalence in order to determine whether the decline in prevalence is adequate and the evaluation area is making satisfactory progress towards elimination. If the survey results show that the prevalence has already fallen to very low levels approaching the elimination thresholds, the evaluation area would move into phase 1B. If the prevalence is still too high, additional phase 1A surveys would be needed at intervals of 3 to 4 years. The results reported here concern only the first round of phase 1A surveys undertaken in APOC countries.

#### Sample population

For each evaluation area some ten sample communities were selected from high risk locations near the river and potential vector breeding sites in that part of the evaluation area where the pre-control endemicity levels were highest according to the results of Rapid Epidemiological Mapping of Onchocerciasis (REMO) [27]. For each sample village, the geographic coordinates were used to obtain the local predicted pre-control prevalence of nodules from the REMO map [3].

#### Parasitological surveys

The parasitological evaluation involved skin snip surveys to estimate the prevalence and intensity of *O. volvulus* infection. All skin-snip surveys were done 11–12 months after the last ivermectin treatment round (thus just before the next distribution). In each surveyed village, all subjects aged 5 years or above who agreed to participate (or whose parents agree for them to participate in the case of children) and who voluntarily presented themselves at the screening centre for the survey were asked for identification data (name, age, sex, number of years resident). In a few surveys that were undertaken before 2011 children between 1 and 5 years of age were also examined but these results for this age group were excluded from the current analysis. For villages with less than 100 examined subjects, the examination results were combined with those for the nearest village if there existed such a village at less than 5 km distance. For very large villages with more than 1000 inhabitants, the surveys were done in a subsection of some 300 inhabitants that was located closest to the river. The surveys used established skin-snip examination methods in which the national onchocerciasis teams had been trained by APOC [32]. Two skin biopsies were obtained (one from each iliac crest) of all individuals who presented themselves for the survey. A 2 mm Holth corneoscleral punch (Storz instrument GMBH, Heidelberg, Germany) was used to obtain the skin biopsies. After each series of two bloodless skin-snip obtained from a subject, the scleral punch was sterilized sequentially in sodium hypochlorite solution, distilled water and then autoclaved by pressure for 15 minutes. The entire process is to ensure that HIV and other blood-borne infections are not transferred. In six evaluation areas where parasitological surveys were undertaken before 2010, the skin snips were microscopically examined after incubation for 30 min in distilled water and a further 24 h in saline for negative skin-snips for the presence and number of *O. volvulus* mf [32]. From 2010 onwards the 30 min reading was dropped and all skin snips were microscopically examined after incubation for 24 h in saline. The numbers of mf were counted and the results recorded for each person examined. Information on the migration history for each person during the last 10 years before the survey was also collected. Finally, exact geographical coordinates were taken for each sample village using a GPS.

#### Comparison with model predictions

The trend in the prevalence of mf after annual ivermectin treatment depends on the pre-control endemicity level and the treatment coverage achieved. Hence, the expected trend in prevalence during the control period will differ between endemicity levels and this had to be taken into account in the interpretation of the survey results. This was done by referring to ONCHOSIM predictions of the expected trends in prevalence for different pre-control endemicity levels and treatment coverage [33, 34]. This model has been used extensively to support policy making in onchocerciasis control [34–37] and model-predicted trends in infection and transmission levels were found to match well to those observed during onchocerciasis control after cessation of control activities [24, 38–40].

**Fig. 2:**
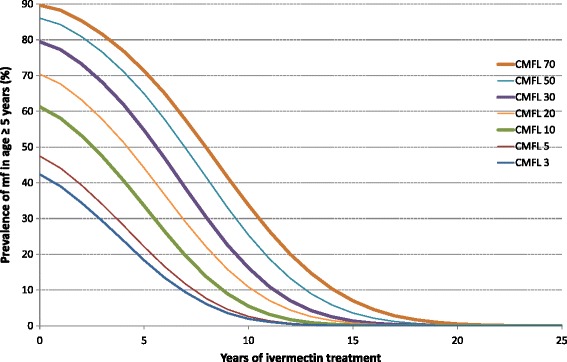
Predicted trends in the prevalence of mf by pre-control endemicity level

The comparison of observed trends and model predictions was done in two ways. Firstly, in order to give a general appreciation of the observed data for each evaluation area in relation to predictions, we plotted the observed mf prevalence for the surveyed villages of each evaluation area against the predicted trends shown in Fig. 2. Figure 2 shows the predicted trends in the prevalence of mf in endemic foci for which the pre-control endemicity levels range from a very low CMFL of 3 mf/snip (mf/s) to a very high CMFL of 70 mf/s and a treatment coverage of 75 % (the average treatment coverage in the evaluation areas). Assumptions underlying these predicted trends are further explained in Additional file 2.

The results are shown in three graphs for three different ranges of pre-control endemicity levels: high (CMFL >30mf/s or prevalence of mf >79 %), moderate (10< CMFL ≤30 mf/s or 61 % < prevalence of mf ≤79 %) and low (CMFL ≤10 mf/s or prevalence of mf ≤61 %). For each surveyed village, the pre-control endemicity level was estimated from the map of the predicted pre-control prevalence of nodules in APOC countries, and converted to the prevalence of mf as described in the statistical classification section below.

Secondly, we undertook a detailed statistical analysis to compare the observed prevalence for each surveyed village and each evaluation area with the corresponding model predictions and classify the decline in prevalence in each evaluation area as “faster than predicted”, “on track” or “delayed” after taking the statistical uncertainty in the various data sources into account, as explained below.

#### Statistical classification of the decline in mf prevalence in an evaluation area

The classification was done in four steps: 1) estimate the pre-control nodule prevalence in adult males (age 20+) from REMO data; 2) translate pre-control nodule prevalences in adult males to pre-control mf prevalences in the general population (age 5+, standardised to the OCP population); 3) predict the expected mf prevalences at the time of the phase 1A survey using ONCHOSIM, given the pre-control mf prevalences from step 2 and the history of control; and 4) given observed and predicted mf prevalences at the time of the Phase 1A survey, calculate the probability that the true decline in infection levels following onchocerciasis control in the evaluation area is faster or slower than predicted by ONCHOSIM. Because each of the four steps involves uncertainty, we used Monte Carlo simulation to propagate all uncertainty to the final estimate in step 4. Below we explain each step in more detail.

In the first step, we determined for each surveyed village location *i* the pre-control prevalence of nodules *Prenod*_*i*_ in adult males (age 20+). For APOC countries, the geographic distribution of pre-control endemicity levels has been previously estimated using a geostatistical analysis of REMO survey results in 14,473 villages, yielding a detailed 1 km resolution map of predicted pre-control prevalence of nodules, including prediction standard error [3]. Using this map, we defined nodule prevalence *Prenod*_*i*_ as a random variate following a normal distribution N(*μ*_nod,*i*_, σ_*i*_^2^), where *μ*_nod,*i*_ is the geostatistical prediction of the pre-control prevalence of nodules at location *i*, and σ_*i*_ is the corresponding prediction standard error. For each location *i* in the evaluation area, we generated 100 000 random draws from the normal distribution N(*μ*_nod,*i*_, σ_*i*_^2^) for use in the second step.

In the second step, we translate pre-control nodule prevalence *Prenod*_*i*_ in adult males to pre-control mf prevalence *Premf*_*i*_ in the general population (age 5+, standardised to the OCP population). For this conversion, we used a Bayesian statistical model, which has been previously fitted to village-level data on pre-control nodule prevalence in adult males and mf prevalence in the general population from several sites in West and Central Africa [41]. We accounted for uncertainty in the conversion from *Prenod*_*i*_ to *Premf*_*i*_ by drawing random values from the joint posterior distribution of parameter values of the conversion model, and using one random set of parameter values for the conversion of each of the 100 000 draws of *Prenod*_*i*_ for each location *i* from step one (for further details see Additional file 3).

In step three, we used ONCHOSIM [33, 36] to predict the mf prevalence in the general population at the time of the phase 1A survey, given pre-control mf prevalence *Premf*_*i*_ from step 2 and the reported history of control. First, we simulated trends in village infection levels in ONCHOSIM for all combinations of a pre-defined set of pre-control infection levels (CMFL 3, 5, 10, 20, 30, 50 and 70 mf/s) and a set of treatment coverage levels (60 %, 65 %, 70 %, 75 %, 80 %). More details regarding methods used and assumptions underlying the ONCHOSIM predictions is provided in Additional file 2. By performing 1 000 repeated stochastic simulations for each combination, we produced a sample from the distribution of predicted mf prevalences levels over time. Then, given a draw of *Premf*_*i*_ from step 2 and the duration and average treatment coverage of CDTi in location *i*, we determined the four closest simulation scenarios (nearest simulated average pre-control mf prevalence below and above *Premf*_*i*_, and nearest simulated treatment coverage below and above the reported treatment coverage), randomly drew one simulation from each of the four simulation scenarios, and performed linear interpolation to produce a draw of *Premf*_*i*_, the predicted mf prevalence at the time of the Phase 1A survey. This was repeated for all 100,000 draws for each location *i*.

Last, in step four, we calculated the difference *D*_*i*_ between the observed and predicted mf prevalences for each location *i* and the mean difference *D* for all surveyed locations in the evaluation area. We then calculated the probability that the observed decline was lower or higher than predicted. To do so, we first quantified the uncertainty in the observed mf prevalence in a Bayesian framework, assuming a uniform prior for the unknown true mf prevalence and a binomial likelihood for the observed data. Under these assumptions, the posterior distribution of the “true” mf prevalence *Truemf*_*i*_ is a Beta distribution with parameters *α* = (*m*_*i*_ + 1) and *β* = (*n*_*i*_ − *m*_*i*_ + 1), where *n*_*i*_ is the number of individuals examined for mf in the surveyed village at location *i* and *m*_*i*_ is the number of individuals that were mf positive [42]. Next, for each of the 100,000 draws of *Predmf*_*i*_ (which include all uncertainty from previous conversion steps), we drew a random value of *Truemf*_*i*_ and calculated the difference *D*_*i*_ between the two, resulting in 100,000 draws for *D*_*i*_ in each location *i*. Finally we calculated the mean difference over all surveyed locations, resulting in 100 000 draws for the mean difference *D* in the evaluation area.

Using the posterior sample of *D*, we calculated the probability p_L_ that *D < 0*, i.e. that the true mean prevalence of mf in the surveyed locations is lower than the model-predicted mean prevalence and the probability p_H_ that *D > 0*, i.e. that the true mean prevalence of mf in the surveyed locations is greater than the model-predicted mean prevalence. The decline in the prevalence of mf in the evaluation area was subsequently classified as (i) significantly faster than predicted (p_L_ >0.975), (ii) delayed: decline significantly slower than predicted (p_H_ >0.975), or (iii) on track (p_L_ ≤0.975 and p_H_ ≤0.975, i.e. the equal tailed 95 % credible interval for *D* includes 0).

### Phase 1B evaluation

#### Objective

To confirm that residual infection and transmission levels are below defined elimination thresholds and that treatment can be safely stopped, epidemiological and entomological evaluations are needed throughout the evaluation area. In this article we report the results of the epidemiological evaluations only. The entomological evaluations are still ongoing and will be reported at a later stage.

#### Sample population

The epidemiological evaluation involved parasitological surveys using the same skin snip methodology as in phase 1A. To ensure that there remained no pockets of infection that could cause recrudescence of transmission after cessation of treatment, Phase 1B surveys were planned such that together with the Phase 1A surveys, they provided complete spatial coverage of all potential transmission areas in the evaluation unit. The framework document [27] recommends that survey villages are selected along the main rivers and affluents at a distance of no more than 20 to 30 km between villages. During the actual evaluations, the selection of villages was generally done at shorter distances and the average distance to the nearest village was 8 km.

#### Criteria for stopping treatment

The conceptual and operational framework document defines as the provisional epidemiological criteria for stopping ivermectin treatment a prevalence of mf <1 % in 90 % of surveyed villages and <5 % in all surveyed villages [27]. The provisional entomological criterion was defined as a vector infectivity rate <0.5 infected fly per 1 000 flies. These criteria were first postulated based on large-scale experiences with cessation of vector control over some 500,000 km^2^ in the OCP countries and ONCHOSIM predictions [35, 43]. At the time of cessation of vector control the prevalence of mf in first line villages was on average 1.4 % with a maximum of 4.8 % but, as predicted by ONCHOSIM, these residual levels of infection did not result in recrudescence of transmission when vector control was stopped [40, 43, 44]. The postulated criteria were subsequently validated for ivermectin-based control in the proof-of-principle study in Mali and Senegal [24] where treatment was stopped based on these criteria and where there was no recrudescence of infection and transmission up to 5 years after cessation of treatment. Based on this empirical evidence, APOC adopted these criteria provisionally for decision-making on stopping ivermectin treatment but noted that they should be revised when further evidence becomes available. Turner et al. [45] and Stolk et al. [29] refer to these epidemiological criteria as the provisional Operational Thresholds for Treatment Interruption followed by Surveillance (pOTTIS) and summarise them for modelling purposes into an overall microfilaria prevalence <1.4 % (the weighted mean of the 2 prevalence thresholds in the framework document definition). In our analysis we also use the threshold of 1.4 % for the overall mf prevalence in the evaluation area. However, the purpose of the pOTTIS was not only to establish that the overall prevalence in the evaluation area was very low but also to ensure that there remained no significant local pockets of infection that could lead to recrudescence. We therefore included in our analysis a second criterion relating to the pOTTIS upper limit prevalence of 5 % and taking into account the sampling strategy used. In phase 1B, sample villages to be surveyed were selected at intervals of less than 20 km along the major rivers and affluents in the evaluation area. Hence, each selected sample village can be regarded as representing a river stretch (8 km length on average, i.e. within the flight range of the vector) and the examined village population as a sample from the population of this river stretch. The sampling method used is then a type of stratified sampling in which each river stretch is a stratum and the population of a surveyed village a random sample of the population of the corresponding stratum. Our second criterion is that the true prevalence of mf for each stratum should be below 5 % or, formulated otherwise, that the maximum stratum prevalence of mf should not exceed 5 %.

#### Analysis of phase 1B evaluation data

For each evaluation area we assessed whether the two criteria for stopping treatment were met after taking the statistical uncertainty in the sample data into account. As for the Phase 1a data, this was done again using a combination of Bayesian statistics and Monte Carlo sampling.

For each stratum *S*_*j*_ in an evaluation area, we define the posterior distribution of the true mf prevalence *Pmf*_*j*_ as a beta distribution with parameters *α* = (*m*_*j*_ + 1) and *β* = (*n*_*j*_ − *m*_*j*_ + 1) [42]. Here, *n*_*j*_ is the number of individuals examined for mf and *m*_*j*_ the number of examined individuals who were mf positive in the survey that was undertaken in stratum *S*_*j*_. Next, we generated draws from the posterior distributions of the true mf prevalence *Pmf* in the evaluation unit and the true maximum stratum prevalence of mf *Pmax* (i.e. the maximum of all *J* strata in the evaluation unit) by generating a random value *Pmf*_*j*_ from Beta(*m*_*j*_ + 1, *n*_*j*_ − *m*_*j*_ + 1) for each of the *J* strata, and calculating the average $\left(Pmf=\frac{1}{J}{\displaystyle {\sum}_{j=1}^J Pm{f}_j}\right)$ and maximum (*Pmax* = max_*j* = 1 : *J*_(*Pmf*_*j*_)) of the *J* draws of *Pmf*_*j*_. This process was repeated 100,000 times to generate probability distributions of *Pmf* and *Pmax*. These probability distributions were subsequently used to calculate the probability that the true area prevalence of mf *Pmf* exceeds 1.4 % and the probability that the maximum stratum prevalence of mf *Pmax* exceeds 5 %.

Each evaluation area was then classified as having met the epidemiological criteria for stopping treatment if the probability of mf prevalence exceeding 1.4 % was under 0.01 and if the probability of the maximum stratum prevalence being over 5 % was less than 0.05. Evaluation areas that did not meet these two criteria were classified as “close to elimination” if the percentage of mf positives in the total sample was less than 1.4 % and as “criteria not met” if the percentage of mf positives was greater than 1.4 %.

#### Overall classification using all available data from phases 1A and 1B

Finally we used all available data to classify the 58 evaluation areas according to their progress towards elimination into four categories: i) meets epidemiological criteria for stopping treatment (same as the phase 1B classification), ii) close to elimination (all other areas with overall prevalence <1.4 %), iii) on track but still some years to go (decline in prevalence equal/faster than predicted but overall prevalence still >1.4 %) and iv) unsatisfactory progress (decline in prevalence delayed and overall prevalence >1.4 %). Table 1 provides for easy reference a summary of the classifications used for the different phases.

**Table 1:** Summary of the classifications used for the different phases

Phase (objective)	Classification	Criteria
Phase 1A (Assess decline in prevalence)	Faster decline	Prevalence declining significantly faster than predicted
	On track	Prevalence not significantly different from predicted
	Delayed decline	Prevalence declining significantly slower than predicted
Phase 1B (Determine if treatment can be stopped)	Stopping criteria met	Area prevalence significantly below 1.4 % and maximum stratum prevalence significantly below 5 %
	Close to elimination	Above thresholds not met but <1.4 % mf positives in sample
	Stopping criteria not met	Above thresholds not met and >1.4 % mf positives in sample
Overall classification using all data (Assess progress towards elimination)	Stopping criteria met	Stopping criteria met in phase 1B
	Close to elimination	Close to elimination in phase 1B or <1.4 % mf positives in phase 1A
	On track but some time to go	Prevalence decline faster/on track and > 1.4 % mf positives in sample
	Unsatisfactory	Prevalence decline delayed and > 1.4 % mf positives in sample

### Implementation of the epidemiological evaluations in APOC countries

The implementation of the epidemiological evaluation was the responsibility of the Ministry of Health of each APOC country in collaboration with its partners in onchocerciasis control and with technical and financial support from APOC. Each APOC country has a National Onchocerciasis Task Force which brings the various partners together in order to coordinate the onchocerciasis control activities in the country. The manager of the National Onchocerciasis Task Force is the responsible officer for onchocerciasis control in the Ministry of Health. The National Onchocerciasis Task Force of each country submitted to APOC a plan of action and budget for epidemiological surveys. Upon approval of this plan, APOC provided the agreed funding and external experts to train and support national teams with the implementation of epidemiological evaluation.

All surveys were reportedly undertaken according to the standard procedures as described in the Epidemiological Evaluation Guide of APOC that is based on the OCP guide for epidemiological evaluations of onchocerciasis control that has been in use in Africa for more than four decades [46]. In almost all surveys there was direct APOC involvement in the actual survey activities in the field especially during the first years by sending technical advisors and/or APOC staff for planning, training and supervision to standardize the quality of the surveys across the countries. All survey results were reviewed by the Technical Consultative Committee of APOC, a group of experts who technically advised the programme. The data was recorded on standard forms and processed at country and APOC level for data cleaning and double data entry before forwarding the data for analysis.

## Results

### Results of phase 1A evaluations

Phase 1A epidemiological evaluations were conducted between 2008 and 2014 in 54 evaluation areas in 12 APOC countries, involving parasitological surveys in 639 sample villages where a total of 127 665 people were examined for the presence of *O. v* mf in the skin (Table 2). Of the 54 evaluation areas, 21 were mesoendemic (maximum pre-control nodule prevalence between 21 and 42 %) and 32 hyperendemic (maximum nodule prevalence >42 %). On average some 12 villages were surveyed per evaluation area. The median number of years of ivermectin treatment with at least 60 % reported treatment coverage was 10 years, ranging from 3 years for two areas in the Democratic Republic of Congo (where ivermectin had been distributed for 8 to 10 years but with insufficient treatment coverage for most years) to 21 years for an area in Cameroon (Touboro) where one of the first community trials of ivermectin was done [47].

**Table 2:** Results of phase 1A surveys and comparison of observed and predicted prevalences for each evaluation area

Country	Evaluation area	Maximum pre-control prevalence of nodules in evaluated villages (%)	Treatment History		Year of evaluation								Comparison with Onchosim model predictions			
			Start year of treatment	Treatment rounds with >60% coverage		Number of villages surveyed	Number of persons examined	Number mf positive	% mf positive	Maximum village prevalence of mf (%)	Maximum village CMFL (mf/s)	Mean village prevalence of mf (%)	Predicted mean prevalence of mf (%)	Probability that true prevalence < predicted prevalence (Pl)	Probability that true prevalence > predicted prevalence (PH)	Progress towards elimination^a^
Burundi	Cibitoke Bubanza	49.1	2005	6	2012	10	3,424	0	0.0	0.0	0.0	0.0	17.5	1.000	0.000	++
Cameroon	Adamawa II	57.5	1999	9	2012	9	2,816	53	1.9	6.8	0.2	1.7	8.3	0.999	0.001	++
	Centre 1	71.9	2001	9	2011	12	2,576	1,377	53.5	71.6	5.3	52.3	31.0	0.000	1.000	−
	Littoral 2	59.9	2000	9	2011	10	1,576	759	48.2	69.4	5.1	48.6	16.5	0.000	1.000	−
	North Tchollire	54.0	1999	10	2010	9	1,377	84	6.1	32.8	3.2	6.8	8.1	0.579	0.421	+
	North Toubouro	81.9	1989	21	2010	10	1,230	48	3.9	12.2	0.3	4.0	1.3	0.000	1.000	−
	South West I	71.7	1999	10	2012	8	2,159	257	11.9	45.5	6.8	14.2	13.3	0.341	0.660	+
	South West II	65.6	2001	8	2012	9	2,055	283	13.8	41.2	5.3	13.2	18.9	0.927	0.073	+
	Western Province	86.4	2001	8	2011	14	3,239	284	8.8	38.6	1.3	9.7	32.0	1.000	0.000	++
CAR	Basse-Kotto	59.7	1999	12	2010	9	2,758	41	1.5	3.8	0.1	1.6	8.6	1.000	0.000	++
	Ouaham Pende	60.1	1999	15	2012	10	2,455	0	0.0	0.0	0.0	0.0	4.9	1.000	0.000	++
	Ouaka	64.6	1999	13	2012	9	2,443	0	0.0	0.0	0.0	0.0	2.3	0.996	0.004	++
Chad	Logon Occidental	37.9	1998	11	2012	10	3,320	0	0.0	0.0	0.0	0.0	0.1	0.239	0.739	+
	Logone Oriental	62.8	1998	11	2012	19	4,403	5	0.1	2.1	0.0	0.1	1.0	0.883	0.117	+
	Mayo Kebbi East	45.2	1998	12	2013	5	1,660	0	0.0	0.0	0.0	0.0	0.1	0.295	0.553	+
	Mayo Kebbi West	44.4	1998	12	2013	18	6,425	1	0.0	0.3	0.0	0.0	0.1	0.227	0.772	+
	Moyen-Chari	40.1	1998	8	2009	9	1,337	0	0.0	0.0	0.0	0.0	3.4	0.997	0.003	++
Congo	Bouenza	28.3	2001	7	2011	10	1,585	13	0.8	5.6	0.0	0.7	8.4	1.000	0.000	++
	Pool	51.0	2004	7	2012	22	3,335	485	14.5	52.3	8.8	15.7	11.6	0.041	0.959	+
DRC	Bas-Congo	65.9	2004	3	2012	22	4,823	2,140	44.4	71.3	9.8	40.5	49.3	0.989	0.011	++
	Sankuru	86.3	2003	3	2011	11	1,494	810	54.2	89.7	14.4	51.8	74.0	1.000	0.000	++
	Uélé	96.4	2002	5	2012	10	1,752	595	34.0	68.4	4.9	33.3	62.5	1.000	0.000	++
Ethiopia	Kafa,Shekka,Bench Maji	47.4	2001	7	2011	10	2,167	111	5.1	19.2	0.4	4.7	19.9	1.000	0.000	++
	North Gondar	31.2	2003	7	2011	10	1,927	0	0.0	0.0	0.0	0.0	9.7	1.000	0.000	++
Liberia	Lofa, Bong, Nimba	27.6	1999	5	2013	19	3,554	244	6.9	18.1	1.0	6.6	17.6	0.999	0.001	++
Malawi	Malawi Extension	21.6	2000	7	2011	9	1,960	0	0.0	0.0	0.0	0.0	4.8	1.000	0.000	++
	Thyolo Muanza	34.1	1999	7	2011	11	2,375	0	0.0	0.0	0.0	0.0	8.2	1.000	0.000	++
Nigeria	Adamawa	51.5	2001	11	2012	10	1,574	0	0.0	0.0	0.0	0.0	0.7	0.523	0.477	+
	Cross river	56.1	1998	11	2009	20	3,583	81	2.3	8.3	0.5	2.4	3.0	0.430	0.570	+
	Ebonyi	60.2	1999	11	2010	11	1,976	0	0.0	0.0	0.0	0.0	3.1	0.999	0.001	++
	Edo, Ondo	36.4	2001	8	2010	7	1,540	422	27.4	54.7	3.3	30.2	9.8	0.000	1.000	−
	Ekiti	22.8	2000	12	2012	10	1,939	36	1.9	12.0	0.5	1.9	0.4	0.003	0.997	−
	Enugu, Anambra	63.0	1999	11	2011	11	2,396	86	3.6	13.7	0.2	3.6	4.5	0.602	0.398	+
	FCT	21.1	1999	14	2013	10	1,385	2	0.1	2.9	0.0	0.3	0.1	0.029	0.966	+
	Kaduna	55.8	1991	17	2008	29	5,988	0	0.0	0.0	0.0	0.0	0.1	0.041	0.959	+
	Kano	26.0	2001	12	2013	12	1,820	0	0.0	0.0	0.0	0.0	0.4	0.262	0.738	+
	Kebbi	21.5	2002	7	2013	7	1,365	0	0.0	0.0	0.0	0.0	3.8	1.000	0.000	++
	Kwara	32.2	1999	13	2013	12	2,013	29	1.4	11.2	0.5	1.3	0.3	0.001	0.999	−
	Niger	28.7	2000	12	2013	15	2,820	0	0.0	0.0	0.0	0.0	0.6	0.459	0.541	+
	Osun	25.6	1998	12	2012	11	1,544	0	0.0	0.0	0.0	0.0	1.5	0.882	0.118	+
	Oyo	35.2	2000	9	2013	11	1,235	6	0.5	6.5	0.2	0.9	3.8	0.925	0.075	+
	Plateau Nassarawa	51.5	1998	14	2012	10	1,912	2	0.1	1.2	0.0	0.1	0.3	0.154	0.846	+
	Taraba	61.8	1998	11	2009	10	1,695	214	12.6	60.8	4.6	16.9	6.3	0.000	1.000	−
	Zamfara	12.8	2001	9	2010	7	1,007	0	0.0	0.0	0.0	0.0	1.2	0.835	0.165	+
Tanzania	Kilosa	25.1	2002	10	2012	10	1,488	37	2.5	19.5	1.2	3.3	1.1	0.011	0.990	−
	Mahenge	78.7	1998	7	2009	10	1,792	176	9.8	21.9	2.2	8.3	43.8	1.000	0.000	++
	Morogoro	35.7	2004	7	2013	20	3,406	169	5.0	33.7	3.4	6.1	8.2	0.727	0.273	+
	Ruvuma	92.3	1999	10	2011	12	2,638	104	3.9	16.2	0.4	4.4	20.2	1.000	0.000	++
	Tanga	35.4	2000	9	2010	10	2,047	6	0.3	2.2	0.1	0.4	4.3	0.999	0.001	++
	Tukuyu	39.7	2001	10	2011	10	1,861	0	0.0	0.0	0.0	0.0	4.2	1.000	0.000	++
	Tunduru	58.0	1995	16	2013	10	1,832	0	0.0	0.0	0.0	0.0	0.3	0.331	0.669	+
Uganda	Kasese	45.5	1998	11	2010	10	1,510	51	3.4	12.1	0.1	3.1	4.6	0.651	0.349	+
	Arua Nebbie	87.3	1999	11	2009	21	3,894	90	2.3	32.1	1.0	3.8	10.5	1.000	0.000	++
	Adjumani Mojo	34.9	1999	11	2010	9	1,180	35	3.0	6.7	0.0	2.5	1.4	0.036	0.964	+
Total						639	127,665	9,136	7.2	89.7	14.4		12.1			

^a^Decline in prevalence of mf: ++ Faster: prevalence declining significantly faster than predicted (p_L_ > 0.975)

+On track: prevalence not significantly lower or higher than predicted (p_L_ < 0.975 and p_H _< 0.975)

-Delayed: prevalence declining significantly slower than predicted (p_H_> 0.975)

**Table 3:** Results of phase 1B surveys and assessment if epidemiological criteria for stopping treatment are met

Country	Evaluation area	Pre-control endemicity (maximum nodule prevalence)			Results of Phase 1B surveys					Results of all surveys combined								
			Phase 1B Evaluation year	No. of treatment rounds with >60 % coverage	No. of villages evaluated	Number examined for mf	mf positives			No. of villages evaluated	Number examined for mf	mf positives				Probability that true prevalence of mf in evaluation area exceeds 1.4 %	Probability that true maximum stratum prevalence of mf exceeds 5 %	Epidemiological criteria for stopping treatment met^a^
							Number	%	Maximum % per village			Number	%	Mean % per village	Maximum % per village			
Burundi	Bururi	31.0	2013	7	20	8,621	2	0.02	0.7	20	8,621	2	0.02	0.03	0.7	0.000	0.000	Yes
	Cibitoke Bubanza	49.1	2013	7	10	3,886	1	0.03	0.3	20	7,310	1	0.01	0.01	0.3	0.000	0.000	Yes
	Rutana	24.6	2013	7	20	7,505	0	0.00	0.0	20	7,505	0	0.00	0.00	0.0	0.000	0.001	Yes
Chad	Logon Occidental	37.9	2014	13	9	2,360	0	0.00	0.0	19	5,680	0	0.00	0.00	0.0	0.000	0.000	Yes
	Logon Oriental	62.8	2014	13	43	9,624	33	0.34	11.9	62	14,027	38	0.27	0.36	11.9	0.000	1.000	Close
	Mandoul	66.1	2013	12	32	12,812	28	0.22	3.4	32	12,812	28	0.22	0.26	3.4	0.000	0.205	Close
	Mayo Kebbi East	45.2	2014	13	25	7,648	0	0.00	0.0	30	9,308	0	0.00	0.00	0.0	0.000	0.006	Yes
	Mayo Kebbi West	44.4	2014	13	16	6,660	0	0.00	0.0	34	13,085	1	0.01	0.01	0.0	0.000	0.000	Yes
	Moyen-Chari	40.1	2012	11	20	5,736	0	0.00	0.0	29	7,073	0	0.00	0.00	0.0	0.000	0.017	Yes
	Tandjile	38.7	2013	12	29	8,414	3	0.04	0.7	29	8,414	3	0.04	0.03	0.7	0.000	0.008	Yes
Ethiopia	North Gondar	47.4	2013	9	20	2,986	0	0.00	0.0	30	4,913	0	0.00	0.00	0.0	0.000	0.009	Yes
Malawi	Malawi Extension	21.6	2012	8	12	2,220	29	1.31	8.7	21	4,180	29	0.69	0.46	8.7	0.012	0.997	Close
	Thyolo Muanza	34.1	2012	8	12	2,105	13	0.62	8.2	23	4,480	13	0.29	0.36	8.2	0.004	0.970	Close
Nigeria	Cross River	56.1	2012	14	22	3,832	275	7.18	25.3	42	7,415	356	4.80	5.10	25.3	1.000	1.000	No
	Ebonyi	60.2	2012	13	7	1,465	45	3.07	11.9	18	3,441	45	1.31	1.06	11.9	0.859	1.000	Close
	Enugu Anambra	63.0	2012	12	14	2,595	49	1.89	12.0	25	4,991	135	2.70	2.29	13.7	1.000	1.000	No
	Kaduna	55.8	2012	21	17	2,600	0	0.00	0.0	46	8,588	0	0.00	0.00	0.0	0.000	0.033	Yes
Tanzania	Tanga	35.4	2012	11	20	3,596	1	0.03	0.5	30	5,643	7	0.12	0.11	1.1	0.000	0.028	Yes
	Tukuyu	39.7	2012	11	17	3,146	0	0.00	0.0	28	5,007	0	0.00	0.00	0.0	0.000	0.042	Yes
	Tunduru	67.5	2013	16	10	1,379	0	0.00	0.0	20	3,211	0	0.00	0.00	0.0	0.000	0.020	Yes
Uganda	Adjumani Mojo	45.5	2012	13	19	4,465	63	1.41	9.7	28	5,645	98	1.74	1.76	9.7	1.000	1.000	No
	Kasese	87.3	2012	14	18	4,981	23	0.46	3.5	28	6,491	74	1.14	1.45	12.1	0.999	1.000	Close
Total					392	108,636	565	0.52	25.3	614	157,840	830	0.53	0.52	25.3			

^a^Criteria met:

Yes: Probability <0.01 that the area prevalence exceeds 1.4 % and probability <0.05 that the maximum sample stratum prevalence exceeds 5 %

Close: Above probability thresholds not met but percentage mf positives <1.4 %

No: Above probability thresholds not met and percentage mf positives >1.4 %

Compared to pre-control endemicity levels, the surveys showed a major decline in infection levels. Overall only 7.2 % of the examined persons were infected, the CMFL was below 10 mf/s in all villages from evaluation areas with more than 3 years adequate treatment (coverage ≥60 %) and in 20 evaluation sites all examined people were skin snip negative.

**Fig. 3:**
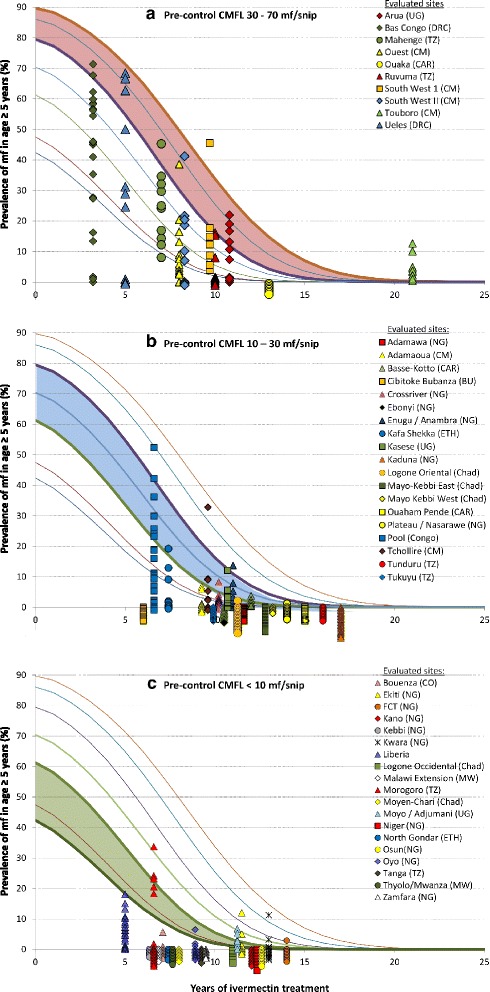
Observed mf prevalences in surveyed villages against predicted trends for three pre-control endemicity ranges: **a**. high (CMFL > 30mf/s), **b**. moderate (10 mf/s < CMFL ≤ 30 mf/s) and c. low (CMFL ≤ 10 mf/s)

In order to give a general appreciation of the observed prevalence data for each evaluation area in relation to model predictions, Fig. 3 shows the observed mf prevalences for the surveyed villages of each evaluation area against the predicted trends. The main results are shown in three graphs for three different ranges of pre-control endemicity levels: high (CMFL >30mf/s), moderate (10< CMFL ≤ 30 mf/s) and low (CMFL ≤10 mf/s). The results for all surveyed villages in an evaluation area are displayed as one cluster in the graph corresponding to the maximum pre-control endemicity level for those villages. If the observed prevalence values for an evaluation area are in agreement with the predictions, the maximum observed prevalence of mf for the surveyed villages is expected to fall within the prevalence range predicted by the model, while the observed prevalences for the other surveyed villages of the evaluation area would have lower values.

Figure 3a show the results for evaluation areas for which the maximum pre-control endemicity level for the surveyed villages was very high. The shaded band shows for such high endemicity levels the range of predicted mf prevalence after annual ivermectin treatment with 75 % treatment coverage. For most evaluation areas, the survey data follow the predicted pattern with the highest prevalences falling within the prediction band, and the prevalences for less endemic villages falling below. There are two exceptions. For the evaluation area of Ouaka in the Central African Republic the observed prevalence was zero in all surveyed villages which was much lower than predicted. In the evaluation area of Touboro in north Cameroon, the prevalence was higher than predicted in several villages.

Figure 3b shows the results for evaluation areas with high endemicity levels (10< CMFL ≤30 mf/s). The overall pattern is as predicted with the maximum prevalence for most evaluation areas within the predicted band and the maximum prevalence declining with number of years of treatment. There are two evaluation areas where the maximum prevalence exceeds the predicted value range. After 12 years of treatment the observed prevalence is zero in nearly all evaluation villages. The main exception to the overall pattern is the result for Cibitoke-Bubanza in Burundi where the prevalence was already zero after only 6 years of treatment, which is much faster than predicted.

Figure 3c shows the results for the lowest endemicity category (CMFL ≤10 mf/s). As predicted, the observed prevalences were generally much lower than in the other two endemicity categories. But there are several sites where the observed prevalence was zero in all surveyed villages after only 7 to 8 years of treatment, i.e. much earlier than predicted. There are also two sites, Ekiti and Kwara in Nigeria, where one village still had a prevalence of about 10 % after 10 years treatment.

**Fig. 4:**
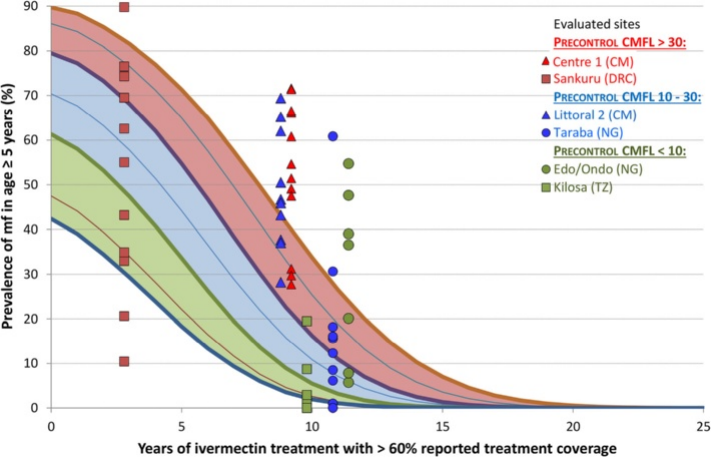
Observed mf prevalences for evaluation areas with evidence that treatment coverage was lower than reported

In addition to the evaluation areas for which the results are shown in Fig. 3, there were six evaluation areas for which there was evidence that treatment coverage was much lower than reported. The results for these sites are shown separately in Fig. 4. In all six sites the observed data reflected the problems with treatment coverage and the observed prevalences were much higher than predicted if treatment coverage had been 75 %. In Centre 1 and Littoral 2 in Cameroon and Edo/Ondo in Nigeria the observed prevalences for most surveyed villages where even higher than the predicted prevalence range. Follow-up investigations in these sites showed much lower treatment coverage than reported and in some years ivermectin had apparently not even reached certain areas. In Taraba there was one major outlier with 60 % prevalence for a village from a small river valley that had been missed in the treatment program. In Kilosa there was a single village with 20 % prevalence and this village had not received treatment during the first 3 years of the CDTi project. The project of Sankuru in the Democratic Republic of Congo only reported >60 % treatment coverage for 3 of the 8 years of its existence but a local treatment coverage survey showed that even in those 3 years the coverage had been significantly lower than reported in half of the villages.

Table 2 shows the results of a detailed statistical comparison of the observed prevalence data with the corresponding predicted prevalences taking the local pre-control endemicity level, treatment coverage and duration of treatment into account. For 23 evaluation areas the decline in the prevalence of onchocerciasis infection was significantly faster than predicted. For another 23 evaluation sites the prevalence of infection was not significantly different from predicted and their progress towards elimination was classified as on track. Eight areas showed delayed results with the prevalence declining significantly slower than predicted. These include five of the evaluation areas with evidence of unsatisfactory treatment coverage shown in Fig. 4 (Centre 1, Littoral, Taraba, Edo/Endo and Kilosa), as well as Touboro in Cameroon (Fig. 3a) and Ekiti and Kwara in Nigeria (Fig. 3c).

### Results of phase 1B evaluations

Phase 1B epidemiological evaluations were conducted in 22 evaluation areas involving skin snip surveys in 392 villages where 108 636 people were examined for onchocerciasis infection (Table 3). For 18 of these evaluation areas, phase 1A results indicated that the prevalence of mf had fallen below the threshold of 1.4 % (14 areas) or was close to the threshold (four areas), justifying progression to phase 1B. In addition, there were four evaluation areas (2 in Burundi and 2 in Chad) where no phase 1A evaluations were done but for which APOC’s Technical Consultative Committee recommended that they move straight into phase 1B given that the phase 1A evaluations in the other evaluation areas in these two countries had shown zero prevalence in all surveyed villages.

The phase 1B surveys were done some 1 to 2 years after the phase 1A surveys, and focused on remaining sections of the evaluation area that had not yet been adequately covered. Hence, the phase 1B surveys are complementary to the phase 1A surveys and we have therefore used the data from phase 1A and 1B combined for the final analysis to determine whether the criteria for stopping treatment were met.

Table 3 shows that 17 out of the 22 evaluation areas meet the first criterion that the probability is <0.001 that the overall prevalence of mf in the evaluation area should exceeds 1.4 %. Only 13 evaluation areas also meet the second criterion of having <0.05 probability that the maximum stratum prevalence of mf exceeds 5 %. Overall, 13 evaluation areas meet both criteria for stopping treatment, three evaluation areas do not meet the criteria and six evaluation areas are classified as “close”, i.e. they do not yet meet the statistical criteria but they are close to elimination as reflected by the fact that the percentage of mf positives in the total sample is less than 1.4 %.

Of the three evaluation areas that did not meet the criteria, the results for Cross River in Nigeria were very disappointing as the promising results from the phase 1A surveys were not confirmed: the overall percentage of mf positives in the phase 1B surveys was 7.2 % (vs. 2.3 % in phase 1A surveys) and one phase 1B village showed a prevalence of 25.3 %. Compared to the model predictions, the 1B prevalences are now even higher than predicted. In the other two evaluation areas the prevalence of mf continued to decline between the two phases but the phase 1B evaluation came too early and some more years of treatment will be needed to achieve elimination.

Table 4 summarises the results of the phase 1A and phase 1B evaluations by country. In 36 evaluation areas only phase 1A evaluations were done and 30 of those showed satisfactory progress towards elimination. This included 14 projects where less than 1.4 % of those examined were mf positive, and these projects were therefore classified as “close to elimination” and ready to proceed to phase 1B. In most of these 14 projects nobody was mf positive and these projects may already have achieved elimination but this requires confirmation in phase 1B surveys. In Burundi, Chad and Malawi all evaluation areas had phase 1B evaluations and all were classified having met the criteria for stopping treatment or being close to elimination. Since these evaluation areas covered all CDTi projects in these countries, nationwide elimination of onchocerciasis may already have been achieved. This is reinforced in Burundi and Chad by results of recent epidemiological assessments for mapping outside the CDTi projects areas that revealed 0 % mf prevalence in all the villages surveyed.

**Table 4:** Classification of the results of phase 1A and phase 1B evaluations by country

Country	Total no. of CDTi projects in the country	Projects evaluated		Evaluation areas with Phase 1A evaluation only				Evaluation areas with phase 1B evaluation			
			Total evaluation areas	Evaluation areas	Close to elimination	On track but still some years to go	Delayed decline in prevalence	Evaluation areas	Meets criteria for stopping treatment?		
									Yes	Close	No
Burundi	3	3	3	0	-	-	-	3	3	0	0
Cameroon	15	8	8	8	0	5	3	0	-	-	-
CAR	1	1	3	3	2	1	0	0	-	-	-
Chad	1	1	7	0	-	-	-	7	5	2	0
Congo	2	2	2	2	1	1	0	0	-	-	-
DRC	20	3	3	3	0	3	0	0	-	-	-
Ethiopia	9	3	2	1	0	1	0	1	1	0	0
Liberia	3	1	1	1	0	1	0	0	-	-	-
Malawi	2	2	2	0	-	-	-	2	0	2	0
Nigeria	27	18	17	13	10	0	3	4	1	1	2
Tanzania	7	7	7	4	0	4	0	3	3	0	0
Uganda	5	3	3	1	0	1	0	2	0	1	1
Total	95	52	58	36	13	17	6	22	13	6	3
	100.0 %	54.7 %	61.1 %	100.0 %	36.1 %	47.2 %	16.7 %	100.0 %	59.1 %	27.3 %	13.6 %

**Fig. 5:**
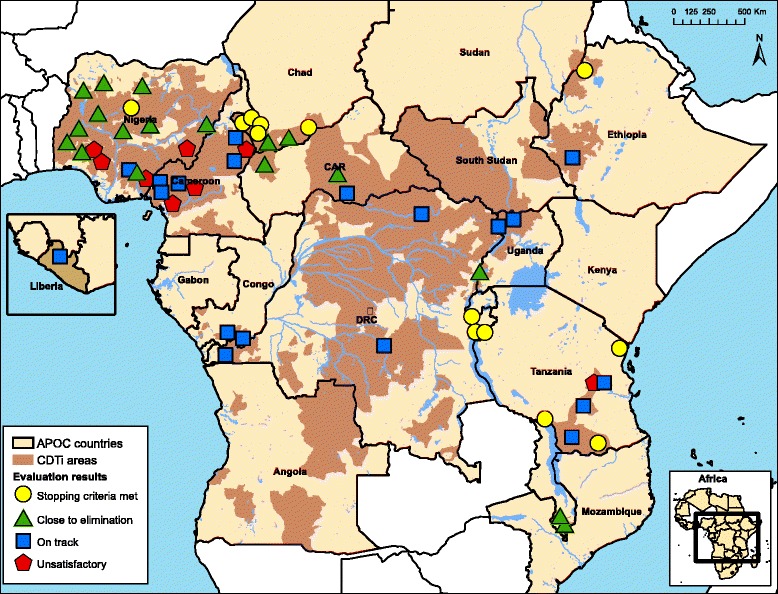
Map of the APOC countries showing the location and classification of the 58 evaluation areas

Table 5 summarises the main conclusions of the epidemiological evaluations. For 13 evaluation areas with a population of 7 million the phase 1B evaluations showed that the epidemiological criteria for stopping treatment had been met. Another 20 evaluation areas with 21 million people are close to elimination. Several of those may already have achieved elimination but this still requires confirmation in phase 1B surveys. Another 18 evaluation areas are on track but still require more years of treatment to reach the elimination breakpoint. Overall 51 of 58 evaluation areas are making satisfactory progress. In seven areas the progress was unsatisfactory, mainly as a result of poor treatment coverage. Figure 5 shows the location of the 58 evaluation areas and the classification of their evaluation results.

**Table 5:** Overall classification of progress towards elimination in all 58 evaluation areas

	Evaluation areas		Population	
Conclusion of the epidemiological evaluation	No.	%	No.	%
1. Satisfactory progress towards elimination				
1.1 Meets criteria for stopping treatment	13	22.4	7,027,252	13.2
1.2. Close to elimination	19	32.8	18,355,590	34.5
1.3 On track but still some years to go	18	31.0	17,397,096	32.7
Subtotal satisfactory progress	50	86.2	42,779,938	80.5
2. Unsatisfactory progress	8	13.8	10,360,552	19.5
Total	58	100.0	53,140,491	100.0

## Discussion

### Control of public health problem

APOC’s original aim was to control onchocerciasis as a public health problem. The results of the epidemiological evaluations in 58 evaluation areas in 12 APOC countries indicate that this has been largely achieved. The infection level when onchocercal blindness starts to become a public health problem has been defined by Dadzie et al. as a CMFL of 10 mf/s [48]. Onchocercal skin disease is also strongly correlated with endemicity [7] but a threshold for its public health importance has not been defined. The epidemiological evaluations showed that the CMFL was below the threshold of 10 mf/s in all surveyed villages in all evaluation areas with more than 3 years treatment. In two thirds of the evaluation areas, the maximum CMFL had even fallen below 0.5 mf/s, i.e. a level where onchocercal blindness is not known to occur [48]. Hence, in all evaluated areas with more than 3 years treatment, including several areas with unsatisfactory treatment coverage, the results indicate that ocular onchocerciasis is no longer a public health problem. Whether the same can be concluded for the 54 ongoing CDTi projects of APOC that have not yet been evaluated is not known. However, most of these remaining projects have had more than 5–7 years ivermectin treatment with good reported coverage and, extrapolating from the results for the evaluated areas, it is likely that onchocerciasis is also no longer a public health problem in those projects. For a few remaining projects with poor treatment coverage, e.g. in Angola, South Sudan and the Democratic Republic of Congo, it is less certain that the disease has been controlled as a public health problem and this requires further evaluation.

### Progress towards elimination

The evaluation results showed that CDTi has achieved much more than the control of a public health problem and that most evaluation areas are making significant progress towards elimination of onchocerciasis infection and transmission. In 13 evaluation areas with a total at-risk population of 7 million people, the epidemiological evaluation data indicate that ivermectin treatment can be stopped. It is noteworthy that in all but one of these areas, the thresholds for stopping treatment were reached after only 7 to 13 years of annual ivermectin treatment, i.e. several years earlier than the 15 to 17 years of annual treatment in the proof-of-principle study in Mali and Senegal [23, 24]. Another 20 areas with 21 million people are classified as being close to elimination. This includes 10 areas with 11 million people for which the phase 1A data suggest that the breakpoint has been reached but which would still require phase 1B evaluations to confirm that this is true for the whole evaluation area. It also includes some projects for which the phase 1B results did not yet meet our strict statistical criteria for elimination but for which the epidemiological results were comparable to those in the proof-of-principle study. In our opinion these areas warrant experimental cessation of treatment, using the same methodology as in the proof-of-principle study, in order to test whether treatment can be safely stopped and strengthen the evidence base for elimination thresholds. Another 15 evaluation areas with 17 million people appear on track to elimination but still require several more years of treatment to reach the elimination thresholds. Overall 51 evaluation areas with 45 million people were making satisfactory progress towards elimination.

Progress was not satisfactory in seven evaluation areas with a population of 8 million people. The main reason was poor treatment coverage which proved to be much lower than reported in five areas as evidenced by the findings of independent coverage surveys or local information obtained by the survey teams. For these areas, the unsatisfactory results concerned all surveyed villages and indicated that the whole area was lagging behind. Major improvements in implementation, supervision and monitoring, and many additional years of treatment will be required to achieve elimination in these areas. The feedback of the unsatisfactory evaluation results to the CDTi projects concerned has already produced dramatic improvements in treatment coverage for several of them.

### Epidemiological modelling

In our analysis we have made extensive use of epidemiological modelling for the interpretation of the epidemiological evaluation data. Onchocerciasis models predict that the declines in the prevalence and intensity of infection during long-term ivermectin treatment programmes depend on the initial local endemicity level and ivermectin treatment coverage [29, 34, 45]. The observed trends in the prevalence of infection in the evaluated areas were consistent with this. The prediction that the decline in infection levels during onchocerciasis control depends on the pre-control endemicity level has been previously confirmed for vector control by the extensive longitudinal evaluation data of the Onchocerciasis Control Programme in West Africa [38]. The current results provide the first large-scale empirical confirmation that the decline in infection levels after ivermectin treatment is also strongly influenced by the initial endemicity levels. In the areas with the highest pre-control endemicity levels, there were, as predicted, still many villages with a prevalence between 10 and 25 % after 10 years of treatment while in the evaluation areas with the lowest endemicity levels the prevalence in nearly all evaluated villages was 0 % after 10 years. Higazi et al. also reported that the prevalence of mf as well as the vector infectivity rate had fallen to insignificant levels after 10 years of ivermectin treatment in a focus with a very low pre-control endemicity level in Sudan where treatment was given annually for the first 8 years and then at 6-monthly intervals [49]. This effect of endemicity level needed to be taken into account in the interpretation of the evaluation results and we have therefore for each surveyed village compared the observed prevalence with the ONCHOSIM predicted prevalence after correcting for the local pre-control endemicity level and treatment coverage and taking statistical uncertainty into account. This has enabled us to identify areas where the epidemiological evaluation results indicated satisfactory progress towards elimination, and areas where the decline in prevalence was slower than predicted.

### Challenges for elimination

One of the areas with a slower-than-predicted decline in prevalence was the Touboro focus in Cameroon for which the highest pre-control nodule prevalence according to the REMO map was 86 % which corresponds to a CMFL of about 70 mf/s. However, during the pre-control period there have been several parasitological studies in this area showing some of the highest recorded onchocerciasis endemicity levels in the world, with a pre-control CMFL as high as 303 mf/s in one village near the border of Chad [47]. After 21 years of annual ivermectin treatment, the average prevalence had fallen to as low as 4 % and the maximum village prevalence and CMFL recorded as part of APOC surveys were only 12 % and 0.27 mf/s in a village where one of the pre-control parasitological surveys had shown a prevalence of 90.5 % and a CMFL 115.9 mf/s. That represents an enormous reduction in infection from the extreme levels of the pre-control period, but it is still significantly higher than predicted for 21 years of annual treatment with 72 % coverage at a pre-control CMFL of 70 mf/s, i.e. the local endemicity level estimated from the nodule prevalence map and the maximum endemicity level considered in the ONCHOSIM predictions. We have therefore done some ad hoc ONCHOSIM simulations for a pre-control CMFL of 115 mf/s which indicated that the observed prevalence data for Touboro after 21 years of ivermectin treatment are still broadly consistent with predictions. But it does raise the question of how many years of treatment would be required in such extremely endemic areas and whether annual treatment can ever achieve complete elimination [50]. In such ‘holo-endemic’ areas the end-game might require alternative strategies such as additional vector control or test and treat strategies [51] where the relatively low number of people remaining infected with *O. volvulus* would be identified by rapid tests such as the LTS DEC-patch [52] followed by skin snips to confirm a positive result and treated with a macrofilaricidal regimen [53]. The analysis has also allowed the identification of areas where the decline in prevalence was faster than predicted. There were several such areas which may indicate that the ONCHOSIM model, as currently quantified, somewhat underestimates the long-term impact of ivermectin treatment.

The residual infection levels in Touboro also pose another challenge. The focus lies along the Vina River, close to the border with Chad. Downstream from the Touboro focus, the Vina River runs along and then across the border into an onchocerciasis area in Logone Oriental in Chad for which the phase 1B evaluation data indicate that it is close to elimination. However, there is still one village with more than 10 % mf prevalence in the evaluation area in Chad and that village lies along the Vina River at 12 km from the border with Cameroon. This situation provides a good example of the challenge of onchocerciasis elimination in border areas. Cross-border foci are common in Africa as rivers often form natural borders between countries while they provide also the breeding sites for the vector in onchocerciasis endemic areas. *Simulium damnosum* s.l. is a powerful fly that can travel many (and sometimes more than 20) kilometres in search of a bloodmeal without respecting national boundaries, and elimination of onchocerciasis in cross-border foci requires that infection levels are brought down below the elimination threshold on both sides of the border before treatment can be stopped. Hence cross-border coordination and synchronisation of treatment and evaluation activities is critical for onchocerciasis elimination in such areas. Cross-border foci are an issue for 23 of the 58 evaluation areas, and they are therefore a major challenge for onchocerciasis elimination.

### Stopping criteria and sampling strategy

For the analysis of the phase 1B data to determine whether treatment could be stopped in an evaluation area we used two statistical criteria that correspond to the provisional epidemiological thresholds for treatment interruption (pOTTIS) in APOC’s conceptual and operational framework of onchocerciasis elimination and that take the sampling methodology into account. The sampling used for the epidemiological evaluations was a spatially stratified sampling method in which sample villages were selected at regular distances along the main rivers and affluents throughout the evaluation area. According to the guidelines, villages were to be selected at distances of no more than 20–30 km between villages. In practice, villages were selected at shorter distances with an average distance of 8 km between neighbouring villages. The rivers were thus divided into sections, or strata, with an average length of 8 km and with one sample village located in the centre. The two statistical criteria that we used to classify each evaluation area were that the estimated area prevalence of mf should be less than 1.4 % (with probability >0.99) and that the estimated maximum stratum prevalence of mf should be less than 5 % (with probability >0.95). Of the 21 areas with phase 1B evaluations, 13 met both of these criteria, 5 failed both criteria and 4 evaluation areas in Malawi and Chad had an estimated area prevalence below 0.5 % but failed the maximum prevalence criterion because of an elevated prevalence (between 3.4 and 11.9 %) for one single phase 1B village in each evaluation area. This illustrates that the criterion of an estimated maximum stratum prevalence less than 5 % with more than 0.95 probability is a very strict criterion that requires the observed prevalences to be very low throughout the evaluation area. Compared to the survey data for the proof-of-principle study in Mali and Senegal, where there were several villages that did not meet the maximum prevalence criterion, this criterion may be too strict. However, we consider it prudent to maintain this criterion for routine use until further evidence becomes available on the safety of stopping treatment in such borderline situations.

One challenge for decision making to stop treatment is to ensure there remain no pockets of residual infection which might lead to recrudescence of transmission. Such pockets could be the result of villages having been missed by the treatment programme or local problems with treatment coverage that were not reported. As illustrated for the four evaluation areas in Malawi and Chad, local pockets of infection could be missed if only the overall prevalence criterion is used. We consider it therefore important to also apply the second criterion for the maximum prevalence and to use a stratified sampling method that takes the transmission characteristics of onchocerciasis into account by focusing on potential high risk locations along rivers and ensuring a regularly spaced sample throughout the evaluation area.

Purposive sampling of villages at high risk locations along rivers has always been an essential part of the sampling strategy for epidemiological evaluations of onchocerciasis control in Africa and we have also applied it in our sampling strategy. Our stratified sampling approach assumes that the survey data for the sample village in the centre of the river stratum represents the highest risk for the stratum. This is true for isolated areas where there is usually only one village per river stratum. In other areas locally available entomological data or remote sensing data on location of river rapids were used to identify high risk locations near potential vector breeding sites within each stratum and select the nearest village for the sample. These villages were likely to represent the maximum prevalence for the stratum which is the information we are interested in. For the remaining areas we selected one of the villages closest to the river (previously referred to as 1st line villages [1]), but there was no additional information available to guide the selection of sample villages from the set of 1st line villages. The length of river strata, with an average of 8 km and an upper limit of 20 km, falls within the active flight range of *Simulium damnosum* s.l. [54] and any significant transmission along the river stretch would most likely be reflected in the survey data for the sample village in the centre of the stratum. However, the prevalence for the surveyed village might in some cases underestimate the stratum prevalence. We therefore recommend that river strata with the highest observed prevalence of mf are also included in the entomological evaluation to ensure that they do not constitute significant pockets of transmission that pose a risk for recrudescence. As mentioned before, the decision to stop treatment requires both epidemiological and entomological evaluations. Because of the need for preparatory activities such as training of entomological technicians and prospection to identify vector collection sites, the entomological evaluation took more time to get started but in 2015 they were operational in several evaluation areas. Though none have yet been completed, the preliminary entomological data appear consistent with the findings of the epidemiological evaluations.

### Recent changes in guidelines for verifying onchocerciasis elimination

WHO recently updated the original guidelines on certification of elimination of human onchocerciasis that were produced in 2001 [55]. The new guidelines document that was published in January 2016 describes the recommended verification process in phase 2 and phase 3, but also addresses criteria and procedures for stopping mass drug administration [56]. It recommends that only entomological evaluation criteria and procedures, as described above and employed by APOC, are used to make the decision to stop treatment. However, when the number of collected blackflies is insufficient, epidemiological evaluation data could be added using the OV16 serology test in children below the age of 10 years (the document notes that the recommendation to use OV16 is based on low certainty of evidence). The aim of this serology testing would be to provide information on exposure to transmission over the preceding 10 years. This differs from the evaluation methodology used by APOC where entomological data are collected to measure transmission but where the epidemiological data measure current infection in the human population. The thresholds also differ. The new guidelines recommend as the threshold for stopping treatment an OV16 prevalence of 0.1 % in children below the age of 10 years but it does not provide a rationale for this figure. An important priority for further modelling research would be to clarify the relationship between different indicators and the risk of recrudescence after stopping treatment, and how this varies with endemicity levels. This would help improve decision-making on when to stop treatment which is critically important: stopping too early may result in recrudescence but stopping too late may be very costly [2]. Another issue is whether a single antibody prevalence from an area-wide random sample of children below the age of 10 years, which is the age group at lowest risk in onchocerciasis, would be able to detect residual pockets of infection. Comparative studies of parasitology and OV16 serology are currently ongoing in areas that are close to elimination, and we hope that the results of these studies will help to clarify this.

## Conclusions

The control of onchocerciasis as a public health problem has been virtually everywhere achieved: in the OCP countries in West Africa [6], in the OEPA countries in the Americas [57] and now in the APOC countries, covering all remaining onchocerciasis endemic countries in Africa. The objective of large-scale ivermectin treatment is changing from onchocerciasis control to onchocerciasis elimination. In the Americas, onchocerciasis elimination with ivermectin treatment has always been considered feasible as most onchocerciasis foci in the Americas were small and circumscribed, and most vector species relatively inefficient [21]. Indeed, 6-monthly or even 3-monthly ivermectin treatment has been able to interrupt transmission in most endemic areas in the Americas and several American countries have recently verified nationwide elimination of onchocerciasis based on the results of post-treatment surveillance over a period of 3 years which showed vector infectivity rates significantly below the threshold of one infective fly per 2 000 flies tested [58–60]. But it has always been doubted that onchocerciasis elimination with ivermectin treatment would be feasible in Africa where onchocerciasis was endemic over vast areas over millions of square kilometres spanning 30 countries, with much higher endemicity levels and highly efficient vectors that are capable of migrating over hundreds of kilometres [21]. This perception started to change following the study in Mali and Senegal which provided the proof-of-principle that onchocerciasis elimination was feasible with both annual and 6-monthly ivermectin treatment in at least some endemic foci in Africa [23, 24]. The question became then to what extent these results could be extrapolated to other areas of Africa. The extensive epidemiological evaluation results reported here for 12 APOC countries have shown that all areas with adequate annual treatment coverage are making satisfactory progress towards elimination and that 33 evaluation areas with a total population of 28 million people are close to, or have already reached, elimination. Hence, onchocerciasis elimination now appears feasible in most, if not all, endemic areas in Africa.

The epidemiological evaluation results show great progress towards elimination and for many APOC countries nationwide onchocerciasis elimination is clearly within reach. However, to achieve this, much remains to be done in terms of epidemiological and entomological evaluations, cross-border coordination of treatment and evaluation activities, acceleration of progress towards elimination in areas that are lagging behind, operational research on potential threats to elimination and alternative treatment strategies for holo-endemic and loiasis-endemic areas etc. Unfortunately, APOC, that has been effectively supporting and coordinating these activities, was closed in December 2015 after having achieved its original objective of establishing sustainable CDTi in all endemic areas and controlling the disease as public health problem. WHO is now putting into place a new structure, the Expanded Special Project for the Elimination of Neglected Tropical Diseases, for coordinated technical support for five neglected tropical diseases in Africa that should also coordinate and support the remaining onchocerciasis elimination activities in Africa [61]. However, it is not clear if this initiative will receive the necessary resources. Now that onchocerciasis control and elimination have come so far, and so close to a definite solution for most onchocerciasis endemic areas, we sincerely hope that the endemic countries will maintain the momentum, sustain high treatment coverage and allocate substantial in-country resources for onchocerciasis elimination activities and that the international community will sustain its long-term commitment to onchocerciasis control so that the job can be properly finished.

## Abbreviations

APOC, African Programme for Onchocerciasis Control; CDTi, community-directed treatment with ivermectin; CMFL, community microfilarial load; mf, microfilaria; mf/s, microfilariae per skin snip; OCP, Onchocerciasis Control Programme in West Africa; pOTTIS, provisional operational thresholds for treatment interruption followed by surveillance; REMO, rapid epidemiological mapping of onchocerciasis

## Additional files

Additional file 1: Multilingual abstracts in the six official working languages of the United Nations. (PDF 732 kb) Additional file 2: ONCHOSIM simulations: brief model description, input assumptions and simulations that were done (DOCX 64 kb) Additional file 3: Conversion from *Prenod*_*i*_ to *Premf*_*i*_ (DOCX 13 kb)
