# Supplementary material for: Progress towards onchocerciasis elimination in the participating countries of the African Programme for Onchocerciasis Control: epidemiological evaluation results
Source: Infect Dis Poverty. 2016 Jun 27;5:66. doi: 10.1186/s40249-016-0160-7 (PMC4924267; doi:10.1186/s40249-016-0160-7)
Supplement: Additional file 1: — Multilingual abstracts in the six official working languages of the United Nations. (PDF 732 kb) [file 40249_2016_160_MOESM1_ESM.pdf]

Translation of the abstract into the six official working languages of the United Nations

التقدم نحو القضاء على مرض العمى النهري في البلدان المشاركة في البرنامج الأفريقي لمكافحة مرض العمى النهري: نتائج التقييم الوبائية

افورك تيكل- هونوراث زوري- ماونكيلا نوما- مايكل بوسينسك- لوككوفينغ- ويلما ستولك- جان ريم.
Afework H. Tekle, Honorath G.M. Zouré, Mounkaila Noma, Michel Boussinesq, Luc E. Coffeng, Wilma A. Stolk, Jan H.F. Remme

ملخص الدراسة

خلفية الموضوع

تم إنشاء البرنامج الأفريقي لمكافحة العمى النهري في عام 1995 للقيام بعلاج يركز على المجتمع بستخدام ايفرمكتين (ivermectin) لضبط مرض العمى النهري الذي يعد مشكلة صحية عامة في 20 بلد افريقي وهم يشكلون 80% من عبئ الأمراض على مستوى العالم. وعندما أظهرت الأبحاث أن العلاج الذي يركز على المجتمع بستخدام ايفرمكتين (ivermectin) فعال للقضاء على عدوى العمى النهري تم اعطاء البرنامج الأفريقي لمكافحة العمى النهري في عام 2008 هدف جديد وهو تحديد المكان والزمان المناسبين لايقاف العلاج بشكل آمن. في هذه الدراسة نقدم تقريراً عن نتائج تقييم هذا الوباء التي تمت بين عام 2008 وعام 2014 لتقييم التقدم نحو القضاء على هذا الوباء في المناطق التي تم العلاج فيها بالتركيز على المجتمع بستخدام علاج ايفرمكتين (ivermectin) لمدة ست سنوات.

منهجية البحث

تمت دراسة بعض العينات من خزعات الجلد في القرى على الخط الأول لهذا المرض لتحديد مدى انتشار الدودة الاسطوانية المكروفيلارية. كان هناك مرحلتين للتقييم. تم تقييم الانحدار في إنتشار المرض في المرحلة A1. وتم مقارنة إنتشارالمرض الملاحظ والذي تم التنبؤ به بعد التصحيح للمستوى الوبائي والتغطية العلاجية. واستخدمت طريقة بيسيان الإحصائية ومحاكاة مونتي كارلو لتصنيف الإنحدار في انتشار المرض كأسرع مما تم التنبؤ به أو كما تم التنبؤ به أو أبطئ من النسبة التي تم التنبؤ بها. حيث ان المرض اقترب من مستوى القضاء عليه وتم إطلاق المرحلة B1 لتحديد امكانية إيقاف العلاج بشكل آمن. وقد تم توسيع أخذ العينات من القرية الى كل المنطقة المشمولة بالعلاج الذي يركز على المجتمع بستخدام ايفرمكتين (ivermectin). وتم تحليل المعلومات ضمن إطار طريقة بيسيان الإحصائية للتأكد إذا ماتم استيفاء معاير التوقف عن العلاج أم لا (معدل الإنتشار الكلي 1.4% > و معدل الإنتشار الأقصى للشريحة 5%>).

النتائج

في المرحلة A1 تم فحص 127,665 شخص من 639 قرية في 54 منطقة. وقد انخفضت نسبة انتشار المرض بشكل كبير. ويعد هذا الإنخفاض في معدل الإنتشار أسرع مما تم التنبؤ به في 23 منطقة وكما تم التنبؤ به في 23 منطقة أخرى وأقل مما تم التنبؤ به في 8 مناطق أخرى. وتم فحص 108,636 شخص في المرحلة B1. شملت هذه العينة 392 قرية في 22 منطقة من ضمنها 13 منطقة إستوفت المعايير الوبائية لإيقاف العلاج. حيث وصلت أو اقتربت 32 منطقة بشكل عام (25.4 مليون شخص) من الوصول الى مرحلة القضاء على المرض و في 18 منطقة (17.4 مليون شخص) كانت على الطريق الصحيح للقضاء على المرض إلا أنها كانت بحاجة لسنوات أطول للعلاج في 8 مناطق (10.4 مليون شخص) حيث كان التقدم بالعلاج غير مرضي وليس كما كان متوقعاً.

الخاتمة

لقد تمت السيطرة على مرض العمى النهري إلى حد كبير كمشكلة صحية عامة. وتم إحراز تقدم كبير نحو القضاء على هذا المرض حيث تم فعلاً تحقيق هذا للملايين من الناس. وقد أصبح القضاء على المرض في معظم البلدان التي هي ضمن البرنامج الأفريقي لمكافحة العمى النهري في متناول اليد.

Translated from English version into Arabic by Randa82, through

TRANSLATORS
WITHOUT BORDERS

基于流行病学评估非洲控制盘尾丝虫病规划项目国家的消除盘尾丝虫病进展

Afework H. Tekle, Honorath G.M. Zouré, Mounkaila Noma, Michel Boussinesq, Luc E. Coffeng, Wilma A. Stolk, Jan H.F. Remme

摘要

引言：非洲控制盘尾丝虫病规划起始于1995年，旨在应用伊维菌素建立基于社区的处置措施（community-directed treatment with ivermectin, CDTi）控制盘尾丝虫病， 20个非洲国家的盘尾丝虫病承担了该病全球80%的疾病负担，是重要的公共卫生问题。研究表明CDTi能最终消除盘尾丝虫病，规划于2008年开始需要决定处理措施在何时何地可以安全地停止实施。本文报告了2008年至2014年在实施CDTi不少于6年的地区的流行病学评价，用于评估规划所取得的进展。

方法：在一线村庄经皮肤查旋盘尾丝虫（*O. volvulus*）微丝蚴确定患病率，评估分为两个阶段。其中，1A阶段评估患病率下降水平，根据流行水平和措施覆盖率进行矫正后，比较现实流行病学调查所得与模型预测的患病率，应用贝叶斯统计学和蒙特卡洛模拟区分两者之间的水平。当患病率达到消除水平，便启动1B评估阶段，考虑是否可以停止实施措施问题，采样延伸到所有CDTi覆盖的村庄。调查数据应用贝叶斯统计学分析，决定是否达到停止标准（整体患病率< 1.4%，且最高患病率<5%）。

结果：在1A阶段，共检测了54个区域中639个村庄的127 665人，患病率显著下降，其中有23个区下降水平快于预期，另外23个与预期差不多，但有8个不如预期水平。在1B阶段，共检测了22个区392个村庄的108 636人，其中有13个区符合停止处理措施的标准。总体而言，32个区（近2 540万人）已达到消除或接近消除；18个区（近1 740万人）按预期取得进展，但需要继续实施措施；而有8个区（近1 040万人）的进展不甚理想。

结论：盘尾丝虫病在很大程度上已经得到控制，针对该病的消除已取得巨大的成就，在非洲控制盘尾丝虫病规划项目国家实现全国范围的消除指日可待。

Translated from English version into Chinese by Yin Jian-hai

CHINESE CENTER FOR DISEASE CONTROL AND PREVENTION
NATIONAL INSTITUTE OF PARASITIC DISEASES

Progrès vers l'élimination de l'onchocercose dans les pays participant au Programme africain de lutte contre l'onchocercose: résultats de l'évaluation épidémiologique

Afework H. Tekle, Honorath G.M. Zouré, Mounkaila Noma, Michel Boussinesq, Luc E. Coffeng, Wilma A. Stolk, Jan H.F. Remme

Résumé
Contexte: Le Programme africain de lutte contre l'onchocercose (APOC) a été créé en 1995 dans le but de mettre en place un traitement à l'ivermectine sous directives communautaires (TIDC) afin d'alléger le fardeau sanitaire de l'onchocercose dans 20 pays d'Afrique qui supportaient 80 % de la charge mondiale de la maladie. Quand des recherches ont montré que le TIDC pouvait, à terme, éradiquer l'onchocercose, on a fixé à l'APOC, en 2008, le but supplémentaire de déterminer quand et où on pouvait sans risque arrêter les traitements. Nous rendons compte des résultats de travaux épidémiologiques réalisés entre 2008 et 2014 afin d'évaluer les progrès vers l'élimination dans les zones de TIDC traitées depuis 6 ans ou plus.
Méthodologie: Des biopsies cutanées superficielles prélevées dans les villages de première ligne ont été analysées afin de déterminer la prévalence des microfilaires *O. volvulus*. L'évaluation s'est déroulée en deux phases. La baisse de la prévalence a été évaluée dans la phase 1A. Après correction tenant compte du niveau d'endémicité et de la couverture par le traitement, les prévalences observées ont été comparées aux prédictions du modèle. Nous avons utilisé des statistiques bayésiennes et une simulation selon la méthode de Monte Carlo pour classifier le déclin de la prévalence en « plus rapide que prévu », « conforme » ou « retardé ». Quand la prévalence se rapprochait des niveaux d'élimination, la phase 1B, visant à déterminer si le traitement pouvant être arrêté sans risques, a été lancée et l'échantillonnage dans les villages a été étendu à toute la zone de TIDC. Les données relevées ont été analysées à l'aide d'un cadre bayésien afin de déterminer si les critères d'arrêt étaient remplis (prévalence totale < 1,4 % et prévalence par strate maximale < 5 %).
Résultats: Dans la phase 1A, nous avons examiné 127 665 personnes dans 639 villages de 54 zones et constaté une baisse spectaculaire de la prévalence, qui était plus rapide que prévu dans 23 zones, conforme aux prédictions dans 23 autres et retardée dans 8. Dans la phase 1B, 108 636 personnes de 392 villages ont été examinées dans 22 zones, dont 13 remplissaient les critères épidémiologiques d'arrêt du traitement. Dans l'ensemble, 32 zones (25,4 millions de personnes) avaient atteint ou presque le niveau d'élimination, 18 (17,4 millions) étaient dans les limites mais avaient encore besoin de quelques années de traitement, et les progrès n'étaient pas satisfaisants dans 8 zones (10,4 millions de personnes).
Conclusions: L'onchocercose a été endiguée et ne constitue plus, dans une grande mesure, un problème de santé publique. De grands progrès ont été faits vers l'élimination, qui s'avère déjà atteinte pour des millions de personnes. Pour la plupart des pays participant à l'APOC, l'élimination de l'onchocercose sur tout le territoire national est à portée de main.

Translated from English version into French by Suzanne Assenat, through

TRANSLATORS
WITHOUT BORDERS

Прогресс в ликвидации онхоцеркоза в странах-участницах Африканской программы по борьбе с онхоцеркозом: результаты эпидемиологической оценки

Эфеворк Г. Текл (Afework H. Tekle), Гонорат Г. М. Зоре (Honorath G.M. Zouré), Мункайла Нома (Mounkaila Noma), Мишел Буссинеск (Michel Boussinesq), Люк Э. Коффенг (Luc E. Coffeng), Вилма А. Столк (Wilma A. Stolk), Жан Г. Ф. Реме (Jan H.F. Remme)

Реферат
Исходная информация: Африканская программа по борьбе с онхоцеркозом (The African Programme for Onchocerciasis Control (APOC)) создана в 1995 году для организации на базе местных общин лечения ивермектином (CDTi) в целях борьбы с онхоцеркозом, представляющим проблему общественного здравоохранения в 20 странах Африки, на которые приходилось 80% глобального бремени болезни. После того, как исследование показало, что применение CDTi, в конечном счете, может ликвидировать инфекцию онхоцеркоза, перед Программой APOC в 2008 году была поставлена дополнительная задача: определить, когда и где можно безопасно прекратить лечение. Нами представлен отчет о результатах эпидемиологических оценок, проведенных за период с 2008 по 2014 годы с целью определения прогресса в ликвидации онхоцеркоза в местах лечения препаратом CDTi в течение шести или более лет.
Методы: Для определения распространенности микрофилярий *O. volvulus* проведено медицинское обследование образцов срезов кожи, взятых в деревнях первой очереди. Процесс анализа состоял из двух фаз. В фазе 1А оценивалось снижение распространенности. После внесения поправок на уровень эндемичности и охват лечением проведено сравнение показателей наблюдаемой распространенности и распространенности, спрогнозированной с помощью моделирования. Для классификации снижения распространенности – быстрее прогноза, в соответствии с прогнозом или медленнее прогноза – использовались статистический метод по Байесу и моделирование по методу Монте-Карло. Когда показатель распространенности достиг уровня ликвидации, началась фаза 1В, в которой определялись сроки безопасного прекращения лечения. Область взятия проб в деревнях была расширена на всю территорию применения препарата CDTi. Для установления факта соблюдения критериев прекращения лечения

(общая распространенность <1,4% и максимальная распространенность по группам <5%), данные исследования анализировались в рамках статистического метода по Байесу.
Результаты: В фазе 1А обследовано 127 665 человек из 639 деревень в 54 областях. Отмечено значительное снижение уровня распространенности. Снижение распространенности было быстрее прогноза в 23 областях, в соответствии с прогнозом еще в 23 областях и медленнее прогноза в 8 областях. В фазе 1В обследовано 108 636 человек из 392 деревень в 22 областях, из которых 13 областей соответствовали эпидемиологическим критериям прекращения лечения. В целом, в 32 областях (24,5 млн человек) достигнут уровень ликвидации инфекции или близкий к ликвидации инфекции; в 18 областях (17,4 млн) лечение шло по плану, но требовалось дополнительно несколько лет; в 8 областях (10,4 млн) прогресс был неудовлетворительным.
Выводы: В целом, онхоцеркоз, как проблема общественного здравоохранения, находится под контролем. В его ликвидации достигнут огромный прогресс, а для миллионов людей это заболевание уже ликвидировано. Ликвидация онхоцеркоза в государственном масштабе для большинства стран-участниц Программы APOC достижима.

Translated from English version into Russian by Tatyana Johnson, through

TRANSLATORS
WITHOUT BORDERS

Progreso hacia la eliminación en los países participantes del Programa Africano para el control de la Oncocercosis: resultados de la evaluación epidemiológica

Afework H. Tekle, Honorath G.M. Zouré, Mounkaila Noma, Michel Boussinesq, Luc E. Coffeng, Wilma A. Stolk, Jan H.F. Remme

Resumen

Antecedentes: El Programa Africano para el control de la Oncocercosis (APOC) fue creado en 1995 para establecer el tratamiento dirigido por la comunidad con ivermectina (CDTi) a fin de controlar la oncocercosis como problema de salud pública en 20 países africanos que tenían el 80% del peso de la enfermedad global. Cuando la investigación demostró que la CDTi puede en última instancia eliminar la infección por oncocercosis, se le otorgó a APOC en 2008 el objetivo adicional de determinar cuándo y dónde se puede detener el tratamiento con seguridad. Informamos los resultados de las evaluaciones epidemiológicas llevadas a cabo desde 2008 hasta 2014 para evaluar el progreso hacia la eliminación en áreas de CDTi con ≥ 6 años de tratamiento.
Metodología: Se realizaron cortes en la piel en muestras de aldeas de primera línea para determinar la prevalencia de *O. volvulus* microfilariae. Hubo dos fases de evaluación. La disminución de prevalencia se evaluó en la fase 1A. Las prevalencias observadas y predichas por el modelo se compararon después de corregir el nivel de endemicidad y la cobertura del tratamiento. Se usaron la estadística bayesiana y la simulación de Monte Carlo para clasificar la disminución en la prevalencia como mas rápida que la predicha, dentro de lo previsto o demorada. Cuando la prevalencia se acercaba a los niveles de eliminación, se lanzó la fase 1B para determinar si el tratamiento podría ser detenido con seguridad. El muestreo en la aldea se extendió a toda el área de CDTi. Se analizaron los datos de la encuesta dentro de un marco bayesiano para determinar si los criterios de detención (prevalencia general < 1,4% y prevalencia de estrato máxima <5%) se cumplían.
Resultados: En la fase 1A, se examinaron 127.665 personas de 639 aldeas en 54 áreas. La prevalencia había disminuido drásticamente. La disminución en la prevalencia fue más rápida que la predicha en 23 áreas, dentro de lo previsto en otras 23 y demorada en 8 áreas. En la fase 1B, se examinaron 108.636 personas en 392 aldeas en 22 áreas de las cuales 13 cumplían con los criterios epidemiológicos para detener el tratamiento. En general, 32 áreas (25,4 millones de personas) habían alcanzado o estaban cerca de la eliminación, 18 áreas (17, 4 millones) estaban dentro de lo previsto pero requerían más años de tratamiento, y en 8 áreas (10,4 millones) el progreso era insatisfactorio.
Conclusiones: La oncocercosis ha sido en gran medida controlada como un problema de salud pública. Ha habido grandes progresos para la eliminación que ya aparece haber sido lograda para millones de personas. Para la mayoría de los países que conforman APOC, la eliminación de la oncocercosis a nivel nacional está al alcance de la mano.

Translated from English version into Spanish by two2tango, through

TRANSLATORS
WITHOUT BORDERS
