# Supplementary material for: Progress towards onchocerciasis elimination in the participating countries of the African Programme for Onchocerciasis Control: epidemiological evaluation results
Source: Infect Dis Poverty. 2016 Jun 27;5:66. doi: 10.1186/s40249-016-0160-7 (PMC4924267; doi:10.1186/s40249-016-0160-7)
Supplement: Additional file 2: — ONCHOSIM simulations: brief model description, input assumptions and simulations that were done (DOCX 64 kb) [file 40249_2016_160_MOESM2_ESM.docx]

Supplementary file 1

ONCHOSIM simulations: brief model description, input assumptions and simulations that were done

This document is a supplement to the following manuscript:

Title: Progress towards onchocerciasis elimination in the participating countries of the African Programme for Onchocerciasis Control: epidemiological evaluation results

Authors: A. Tekle, H. Zouré, M. Noma, M. Boussinesq, L.E. Coffeng, W.A. Stolk, J.H.F. Remme

Contents

[1. The ONCHOSIM model 2](#_Toc445134653)

[2. ONCHOSIM input: Probability distributions, functions and parameter values 2](#_Toc445134654)

[3. Design of simulation experiment 7](#_Toc445134655)

[4. References 8](#_Toc445134656)

# The ONCHOSIM model

ONCHOSIM is an established epidemiological mathematical mode**l** for simulating transmission and control of onchocerciasis [[1](#_ENREF_1)], developed by Erasmus MC in collaboration with the Onchocerciasis Control Programme in West Africa (OCP). It has been used extensively to support decision making in onchocerciasis control programs in Africa [[2](#_ENREF_2),[3](#_ENREF_3),[4](#_ENREF_4),[5](#_ENREF_5),[6](#_ENREF_6),[7](#_ENREF_7),[8](#_ENREF_8),[9](#_ENREF_9),[10](#_ENREF_10),[11](#_ENREF_11)]. ONCHOSIM is an individual-based model, describing the transmission of onchocerciasis between individuals in a dynamic human population and the life course and mf production of individuals within the human hosts. The software tracks changes in infection intensity (number of worms, density of mf in the skin) within human individuals over time. Together, the individuals form a dynamic human population that changes in composition over time due to birth and death of individuals; in addition, random outmigration of individuals may be assumed to keep the total population size more or less constant. Transmission of infection by flies is simulated deterministically, taking account of differences between individuals in the exposure to fly bites (related to age and sex and an index representing other personal factors); due to this exposure heterogeneity, the rate of acquisition of new infections and resulting infection intensity vary between human individuals, as does their contribution to the infection pool in flies.

The model can simulate the impact of mass treatment with ivermectin and vector control on transmission and infection indicators. Epidemiological surveys are simulated to obtain information on the infection status of individuals in the population at specified moments in time (model output). Default output includes the mean mf count in two skin snips, summarized at population level in terms of mf prevalence and the community microfilarial load (CMFL) [[12](#_ENREF_12)]. The model accounts for imperfect sensitivity of the skin snip microscopy method. All individuals in the population are assumed to participate in the surveys.

ONCHOSIM is a disease-specific model-variant of the generic WORMSIM modelling framework. For the current project, we used version 2.58Ap9 of WORMSIM. This version of the software has been made available elsewhere, along with a detailed formal description of the model, the program code, and a complete overview of model assumptions and parameter quantifications [[11](#_ENREF_11)].

# ONCHOSIM input: Probability distributions, functions and parameter values

The parameter quantification was largely the same as in our previous analysis [[11](#_ENREF_11)]. For completeness, the table below provides a complete overview of all input assumptions and parameter values, along with references to the source of the information.

Table 1. WORMSIM quantification used in the current study to simulate onchocerciasis transmission. Given that WORMSIM is a general modelling framework that covers various helminthic infections, certain parameters do not apply to onchocerciasis transmission but are listed anyway for the sake of completeness (indicated where applicable).

| **Parameter** | **Value** | | **Source** |
| --- | --- | --- | --- |
| **Human demography** | | | |
| *Cumulative survival (*$F\left( a \right)$*), by age (see also Figure A1)* | | | [[13](#_ENREF_13)] |
| 0 | 1.000 | |  |
| 5 | 0.804 | |  |
| 10 | 0.772 | |  |
| 15 | 0.760 | |  |
| 20 | 0.740 | |  |
| 30 | 0.686 | |  |
| 50 | 0.509 | |  |
| 90 | 0.000 | |  |
| *Fertility rate per woman (*$R\left( a \right)$*), by age* | | | [[13](#_ENREF_13)] |
| 0–14 | 0.000 | |  |
| 15–29 | 0.109 | |  |
| 30–49 | 0.300 | |  |
| 50+ | 0.000 | |  |
| Population trimming | 10% if population size exceeds 440. | | Assumption |
| **Transmission of infection** | | | |
| *General transmission parameters* |  | |  |
| Relative biting rate (*rbr*) | Varied between simulations to modify the annual biting rate. | |  |
| Overall exposure rate of human hosts to central reservoir of infection ($\zeta$) | Not applicable to onchocerciasis transmission ($\zeta=1$) | |  |
| Seasonal variation in contribution to reservoir (*mbr*) | We assumed perannual transmission, with some seasonality. The monthly biting rates for January-December were 104%, 91%, 58%, 75%, 75%, 66%, 102%, 133%,117%, 128%, 146%, and 105% times the average monthly biting rate | | [[14](#_ENREF_14)] |
| Transmission probability (*v*), i.e. the probability that an infective particle in the reservoir successfully develops into a parasite life stage that is capable of infecting a human host | $v=0.07345$; see reference for the derivation of this value, given parameters for fly biology and development of infective L3 larvae within the fly. | | [[5](#_ENREF_5)] |
| Success ratio (*sr*) | $sr=0.31\%$ | | [[15](#_ENREF_15),[16](#_ENREF_16)] |
| Zoophily (*z*) | $z=0.04$. | | [[17](#_ENREF_17)], expert opinion (OCP entomologists) |
| *Individual relative exposure to flies* |  | |  |
| Variation in by age and sex (*Exa*) | Zero at birth, linearly increasing between ages 0–20 from 0 to 1.0 for men and from 0 to 0.7 for women, and then constant from the age of 20 years onwards | | [[15](#_ENREF_15)] |
| Variation due to personal factors (fixed through life) given age and sex ($\alpha_{Exi}$) | Gamma distribution with mean 1.0 and shape and rate varied between simulations | | [[15](#_ENREF_15)], unpublished data from OCP |
| *Individual relative contribution to infection in the fly population* | | | |
| Variation by age and sex (*Coa*) | $Coa=Exa$; individual contribution and exposure to the cloud are perfectly correlated, given they are governed by the same fly bites. | | Assumption |
| Variation due to personal factors (fixed through life) given age and sex ($\alpha_{Coi}$) | $Coi=Exi$; individual contribution and exposure to the cloud are perfectly correlated, given they are governed by the same fly bites. | | Assumption |
| *Host immunity to incoming infections* | | |  |
| Average impact of host immunity ($\alpha_{Imm})$ | Assumed irrelevant for onchocerciasis, hence $\alpha_{Imm}=0$; i.e. no effect of immunity on incoming infections. | | Assumption |
| Immunological memory ($\beta_{Imm})$ | Irrelevant given that $\alpha_{Imm}=0$. | | Assumption |
| **Life history and productivity of the parasite in the human host** | | | |
| Average worm lifespan (*Tl*) | 10 years | | [[6](#_ENREF_6)] |
| Variation in worm lifespan | Weibull distribution with shape 3.8. | | Assumption [[6](#_ENREF_6)] |
| Prepatent period (*pp*) | 1 year | | [[6](#_ENREF_6)], which refers to [[18](#_ENREF_18),[19](#_ENREF_19)] |
| Age-dependent microfilaria production capacity (*R(a)*) | *R(a)* = 1 for 0 ≤ *a* < 5 | | [[6](#_ENREF_6)], which refers to [[20](#_ENREF_20),[21](#_ENREF_21)] |
|  | *R(a)* = 1-((a-5)/15) for 5 ≤ *a* < 20 | |  |
|  | *R(a)* = 0 for *a* > 20 | |  |
|  |  | |  |
| Longevity of microfilariae within host (*Tm*) | 9 months | | [[15](#_ENREF_15)] |
| Mating cycle (*rc*) | 3 months | | [[15](#_ENREF_15)], which refers to [[22](#_ENREF_22),[23](#_ENREF_23)] |
| Male potential (*pot*) | 100 female worms. | | [[15](#_ENREF_15)] |
| *Density-dependent female worm reproductive capacity* |  | |  |
| Worm contribution to host load of infective material ($O(.)$) | 7.6 mf/worm | | [[15](#_ENREF_15)] |
| Exponential saturation of individual female worm productivity per worm present in host ($\lambda_{z}$) | $\lambda_{z}=0$, i.e. no exponential saturation. | | Assumption |
| **Morbidity** | | | |
| Disease threshold (*Elc*) for blindness | Weibull distribution with mean 10.000 and shape 2.0 | | [[3](#_ENREF_3)] |
| Reduction in remaining life expectancy due to blindness (*rl*) | 50% | | [[3](#_ENREF_3)], which refers to partly published data from OCP [[24](#_ENREF_24)]; and [[1](#_ENREF_1)], which refers to [[25](#_ENREF_25),[26](#_ENREF_26)] |
|  |  | |  |
| **Infection dynamics in the cloud** | | | |
| Cloud uptake of infectious material ($U\left( . \right)$) | Exponential saturating function with parameters *a* = 1.2, *b* = 0.0213, and *c* = 0.0861 (see appendix II for the definition of an exponential saturating function). | | [[7](#_ENREF_7)], which refers to [[27](#_ENREF_27),[28](#_ENREF_28)] |
| Monthly cumulative survival of infective material in the central reservoir ($\psi$) | 0%; i.e. the cloud represents a cloud of vectors that transmit infection within the same month. | | Assumption |
| **Mass treatment coverage** | | | |
| Coverage ($C_{w}$) | User-defined. | |  |
| *Relative compliance (*$c_{r}\left( k,s \right)$*) by age and sex (descriptive label used in graphs)* | | | Based on unpublished OCP data |
| age-group | cr(k,males) | cr(k,females) |  |
| 0-4 | 0 | 0 |  |
| 5-9 | 0.75 | 0.5 |  |
| 10-14 | 0.8 | 0.7 |  |
| 15-19 | 0.8 | 0.74 |  |
| 20-29 | 0.7 | 0.65 |  |
| 30-49 | 0.75 | 0.7 |  |
| 50+ | 0.8 | 0.75 |  |
| **Effects of treatment with ivermectin** | | | |
| Proportion of microfilariae cleared from host | 100% | | [[8](#_ENREF_8)] |
| Duration of temporary reduction in female reproductive capacity (${Tr}_{0}$), average | 11 months | | [[8](#_ENREF_8)] |
| Permanent reduction in female worm reproductive capacity ($d_{0}$), average | 34.9% | | [[8](#_ENREF_8)] |
| Proportion of adult worms killed ($m_{0})$ | 0% | | [[8](#_ENREF_8)] |
| Relative effectiveness (*v*) | Weibull distribution with mean 1 and shape 2 | | [[8](#_ENREF_8)] |
| **Vector control** | | | |
| Timing | Not used. | |  |
| Coverage | Not used. | |  |
| **Surveys** |  | |  |
| Dispersal factor for worm contribution to measured density of infective material (*d*) | Exponential distribution with mean 1 | | [[6](#_ENREF_6)] |
| Variability in measured host load of infective material (eggs per gram faeces) | Poisson distribution with mean $ss\left( t \right)$ | | [[6](#_ENREF_6)] |

# Design of simulation experiment

We simulated expected trends in mf prevalence in the population aged 5 years and above during mass ivermectin treatment, for different settings (varying with respect to local transmission conditions and hence community microfilarial load, see table 2) and under alternative scenarios on ivermectin mass treatment (in each scenario we simulated 25 annual rounds of ivermectin mass treatment; the coverage was varied between scenario 60%, 65%, 70%, 75% and 80%. The prevalence and intensity of mf were recorded annually, just before the scheduled treatment round. The dynamic changes between treatment rounds are therefore not visualized in the simulation output.

Because many processes simulated in ONCHOSIM involve probabilities, repeated model simulations based on the same assumptions will results in slightly different predictions because of stochastic variation. Therefore, to assess the average trends and the stochastic variation between simulation runs, we did 1,000 repeated simulation runs per setting and treatment scenario.

In general, before starting simulation of interventions in ONCHOSIM, a 200-year warm-up period is simulated, such as to allow the human and worm population to establish equilibrium levels, given the parameters for average fly biting rate and inter-individual variation in exposure to infection and human population demographics. At the start of the warm-up period, an artificial force of infection is simulated for a user-defined number of years, allowing worms to establish themselves in the human population (here: 4 worms per person per year for 7.5 years). After the 200 warm-up years, the simulated infection levels are no longer correlated with the initial conditions at the start of the warm-up period. Due to chance processes during the warming-up period, it can happen that infection goes to extinction during the warming-up period, especially when the biting rate is relatively low. In this analysis, we discarded all runs where mf prevalence was <25% after the 200-year warming-up period (about 73% and 32% of simulation runs were discarded for settings with CMFL = 3 or CMFL = 5 respectively). These discarded runs were replaced by others until we had 1,000 successful runs for the analysis.

We refer to the main text of the article for a further description of how simulation results were used in the analysis.

**Table 2. Simulated settings: assumptions on biting rate and exposure heterogeneity and model-predicted average CMFL and mf prevalence**

| Pre-set value of CMFL | Relative biting rate ^a^ | Corresponding annual biting rate ^a^ | Value of parameter ^b^  $\boldsymbol{\alpha}_{\boldsymbol{Exi}}$ | Simulated average CMFL | Simulated average mf prevalence in the population aged 5 years and above |
| --- | --- | --- | --- | --- | --- |
| 3 | 0.292 | 9,008 | 3,834 | 3.5 | 40.8% |
| 5 | 0.303 | 9,348 | 3,860 | 4.8 | 46.4% |
| 10 | 0.329 | 10,150 | 3,926 | 10.0 | 61.3% |
| 20 | 0.381 | 11,754 | 4,064 | 19.9 | 73.3% |
| 30 | 0.434 | 13,389 | 4,213 | 29.9 | 79.5% |
| 50 | 0.538 | 16,597 | 4,546 | 50.1 | 86.1% |
| 70 | 0.643 | 19,837 | 4,937 | 69.9 | 89.7% |

^a^ In the simulation, the annual number of fly bites received by adult male person per year is calculated as the sum of monthly biting rates for Jan-Dec for a reference location (usually varying between months, reflecting seasonality of transmission) multiplied by the relative biting rate (representing the mean exposure in adult males in the simulated setting, relative to the exposure in the reference location). The annual biting rate for the reference location was set at 30,850 fly bites per person per year. See table 1 for assumptions regarding seasonality.

^b^ Higher inter-individual variation in exposure to fly bites leads to stronger overdispersion of infection, i.e. higher parasite concentrations in a few often bitten individuals, and lower parasite concentrations in all other, less often bitten individuals. We mimic this by letting the value of $\alpha_{\mathrm{Exi}}$ increase with the annual biting rate.

# References

1. Plaisier AP, van Oortmarssen GJ, Habbema JDF, Remme J, Alley ES (1990) ONCHOSIM: a model and computer simulation program for the transmission and control of onchocerciasis. Comput Methods Programs Biomed 31: 43-56.

2. Alley ES, van Oortmarssen GJ, Boatin BA, Nagelkerke NJD, Plaisier AP, et al. (2001) Macrofilaricides and onchocerciasis control, mathematical modelling of the prospects for elimination. BMC Public Health 1.

3. Coffeng LE, Stolk WA, Zoure HG, Veerman JL, Agblewonu KB, et al. (2013) African Programme For Onchocerciasis Control 1995-2015: model-estimated health impact and cost. PLoS Negl Trop Dis 7: e2032.

4. Coffeng LE, Stolk WA, Zoure HG, Veerman JL, Agblewonu KB, et al. (2014) African programme for onchocerciasis control 1995-2015: updated health impact estimates based on new disability weights. PLoS Negl Trop Dis 8: e2759.

5. Coffeng LE, Stolk WA, Hoerauf A, Habbema D, Bakker R, et al. (2014) Elimination of African onchocerciasis: modeling the impact of increasing the frequency of ivermectin mass treatment. PLoS One 9: e115886.

6. Plaisier AP, van Oortmarssen GJ, Remme J, Habbema JD (1991) The reproductive lifespan of *Onchocerca volvulus* in West African savanna. Acta Trop 48: 271-284.

7. Plaisier AP, van Oortmarssen GJ, Remme J, Alley ES, Habbema JD (1991) The risk and dynamics of onchocerciasis recrudescence after cessation of vector control. Bull World Health Organ 69: 169-178.

8. Plaisier AP, Alley ES, Boatin BA, Van Oortmarssen GJ, Remme H, et al. (1995) Irreversible effects of ivermectin on adult parasites in onchocerciasis patients in the Onchocerciasis Control Programme in West Africa. J Infect Dis 172: 204-210.

9. Plaisier AP, Alley ES, van Oortmarssen GJ, Boatin BA, Habbema JDF (1997) Required duration of combined annual ivermectin treatment and vector control in the Onchocerciasis Control Programme in west Africa. Bull World Health Organ 75: 237-245.

10. Winnen M, Plaisier AP, Alley ES, Nagelkerke NJ, van Oortmarssen G, et al. (2002) Can ivermectin mass treatments eliminate onchocerciasis in Africa? Bull World Health Organ 80: 384-391.

11. Stolk WA, Walker M, Coffeng LE, Basanez MG, de Vlas SJ (2015) Required duration of mass ivermectin treatment for onchocerciasis elimination in Africa: a comparative modelling analysis. Parasit Vectors 8: 552.

12. Remme J, Ba O, Dadzie KY, Karam M (1986) A force-of-infection model for onchocerciasis and its applications in the epidemiological evaluation of the Onchocerciasis Control Programme in the Volta River basin area. Bull World Health Organ 64: 667-681.

13. United Nations Department of Economic and Social Affairs Population Division (2013) World Population Prospects: the 2012 revision, Volume I: Comprehensive Tables.

14. Alley ES, Plaisier AP, Boatin BA, Dadzie KY, Remme J, et al. (1994) The impact of five years of annual ivermectin treatment on skin microfilarial loads in the onchocerciasis focus of Asubende, Ghana. Trans R Soc Trop Med Hyg 88: 581-584.

15. Plaisier AP (1996) Modelling onchocerciasis transmission and control [PhD Thesis]. Rotterdam, the Netherlands: Erasmus University Rotterdam. 181 p.

16. Duke BO (1993) The population dynamics of Onchocerca volvulus in the human host. Trop Med Parasitol 44: 61-68.

17. Habbema JDF, Oortmarssen GJ, Plaisier AP (1996) The ONCHOSIM model and its use in decision support for river blindness control. In: Isham V, Medley G, editors. Models for infectious human diseases - their stucture and relation to data. Cambridge: Cambridge University Press. pp. 360–380.

18. Duke BO (1980) Observations on Onchocerca volvulus in experimentally infected chimpanzees. Tropenmed Parasitol 31: 41-54.

19. Prost A (1980) [Latency period in onchocerciasis]. Bull World Health Organ 58: 923-925.

20. Albiez EJ (1985) Calcification in adult Onchocerca volvulus. Trop Med Parasitol 36: 180-181.

21. Karam M, Schulz-Key H, Remme J (1987) Population dynamics of Onchocerca volvulus after 7 to 8 years of vector control in West Africa. Acta Trop 44: 445-457.

22. Schulz-Key H, Karam M (1986) Periodic reproduction of *Onchocerca volvulus*. Parasitol Today 2: 284-286.

23. Schulz-Key H (1990) Observations on the reproductive biology of Onchocerca volvulus. Acta Leiden 59: 27-44.

24. Dadzie KY, Remme J, Rolland A, Thylefors B (1986) The effect of 7-8 years of vector control on the evolution of ocular onchocerciasis in West African savanna. Trop Med Parasitol 37: 263-270.

25. Prost A, Vaugelade J (1981) [Excess mortality among blind persons in the West African savannah zone]. Bull World Health Organ 59: 773-776.

26. Kirkwood B, Smith P, Marshall T, Prost A (1983) Relationships between mortality, visual acuity and microfilarial load in the area of the Onchocerciasis Control Programme. Trans R Soc Trop Med Hyg 77: 862-868.

27. World Health Organization (1989) Onchocerciasis Control Programme in West Africa: report of the annual OCP research meeting, 20-24 March 1989. (unpublished).

28. Phillipon B (1977) Étude de la transmission d’*Onchocerca volvulus* (Leuckart, 1893) (Nematoda, Onchocercidae) par *Simulium damnosum* Theobald, 1903 (Diptera, Simuliidae) en Afrique tropicale. . Paris: O.R.S.T.O.M.
