# Supplementary material for: Progress towards onchocerciasis elimination in the participating countries of the African Programme for Onchocerciasis Control: epidemiological evaluation results
Source: Infect Dis Poverty. 2016 Jun 27;5:66. doi: 10.1186/s40249-016-0160-7 (PMC4924267; doi:10.1186/s40249-016-0160-7)
Supplement: Additional file 3: — Conversion from Prenod i to Premf i (DOCX 13 kb) [file 40249_2016_160_MOESM3_ESM.docx]

Supplementary file 2

Conversion from ${Prenod}_{i}$ to ${Premf}_{i}$

This document is a supplement to the following manuscript:

Title: Progress towards onchocerciasis elimination in the participating countries of the African Programme for Onchocerciasis Control: epidemiological evaluation results

Authors: A. Tekle, H. Zouré, M. Noma, M. Boussinesq, L.E. Coffeng, W.A. Stolk, J.H.F. Remme

Statistical details for the conversion from ${Prenod}_{i}$ to ${Premf}_{i}$

We accounted for uncertainty in the conversion from ${Prenod}_{i}$ to ${Premf}_{i}$ by drawing random values from the joint posterior distribution of parameter values of the conversion model, and using one random set of parameter values for the conversion of each of the 100,000 draws of ${Prenod}_{i}$ for each location *i* from step one (for further details see supplementary file 1). In short, the distribution of ${Premf}_{i}$, conditional on ${Prenod}_{i}$, was defined in terms of multivariate (MVN) and univariate (N) normal distributions, parameterised in terms of mean(s) and (co)variance: $\text{logit}\left( {Premf}_{i} \right)\sim N\left( \beta_{\text{mf}}^{*}+\frac{\sigma_{\text{mf}}}{\sigma_{\text{nod}}}\cdot\rho_{\text{mf,nod}}\cdot\left( logit\left( {Prenod}_{i\text{,corrected}} \right)-\beta_{\text{nod}}^{*} \right),\left( 1-\rho_{\text{mf,nod}}^{2} \right)\cdot\sigma_{\text{mf}}^{2} \right)$ and $\begin{matrix} \beta_{\text{mf}}^{*} \\ \beta_{\text{nod}}^{*} \end{matrix}\sim MVN\left( B_{\text{region}},\Sigma_{\text{region}} \right)$

Here, $\begin{matrix} \beta_{\text{mf}}^{*} \\ \beta_{\text{nod}}^{*} \end{matrix}$ are the unknown regional mean mf and nodule prevalences with covariance $\Sigma_{\text{region}}$ and mean $B_{\text{region}}$ (in the original data from West and Central Africa). The parameters $\sigma_{\text{mf}}$, $\sigma_{nod}$, and $\rho_{\text{mf,nod}}$ together define the correlation and covariance of mf and nodule prevalence at the village level (within regions). ${Prenod}_{i\text{,corrected}} =\frac{{Prenod}_{i} +\text{Specificity}_{\text{nod}}-1}{\text{Specificity}_{\text{nod}}}$ where the parameter $\text{Specificity}_{\text{nod}}$ represents the specificity of nodule palpation, and was used to correct predicted nodule prevalence ${Prenod}_{i}$ to the “true” nodule prevalence ${Prenod}_{i\text{,corrected}}$.
